# Plant growth-promoting rhizobacteria enhance active ingredient accumulation in medicinal plants at elevated CO_2_ and are associated with indigenous microbiome

**DOI:** 10.3389/fmicb.2024.1426893

**Published:** 2024-08-26

**Authors:** Charles Wang Wai Ng, Wen Hui Yan, Yi Teng Xia, Karl Wah Keung Tsim, Justin Chun Ting To

**Affiliations:** ^1^Department of Civil and Environmental Engineering, The Hong Kong University of Science and Technology, Kowloon, Hong Kong SAR, China; ^2^Division of Life Science and Centre for Chinese Medicine, The Hong Kong University of Science and Technology, Kowloon, Hong Kong SAR, China; ^3^State Key Laboratory of Marine Pollution, School of Energy and Environment, City University of Hong Kong, Kowloon, Hong Kong SAR, China; ^4^Department of Biology, The University of Western Ontario, London, ON, Canada

**Keywords:** PGPR, elevated CO_2_, active ingredient, microbial community, biological pathways

## Abstract

**Introduction:**

Plant growth-promoting rhizobacteria (PGPR) and elevated CO_2_ (eCO_2_) have demonstrated their individual potential to enhance plant yield and quality through close interaction with rhizosphere microorganisms and plant growth. However, the efficacy of PGPR under eCO_2_ on rhizosphere microbiome and, ultimately, plant yield and active ingredient accumulation are not yet fully understood.

**Methods:**

This study investigated how the medicinal plant *Pseudostellaria heterophylla* (*P. heterophylla*) and its rhizosphere microbes respond to PGPR (*Bacillus subtilis* and *Pseudomonas fluorescens*) at eCO_2_ (1,000 ppm).

**Results and Discussion:**

It was found that the yield and active ingredient polysaccharides accumulation in the tuber of *P. heterophylla* were significantly increased by 38 and 253%, respectively. This promotion has been associated with increased root development and changes in the indigenous microbial community. Metagenomics analysis revealed a significant reduction in pathogenic *Fusarium* abundance in the rhizosphere. Potential biocontrol bacteria *Actinobacteria* and *Proteobacteria* were enriched, especially the genera *Bradyrhizobium* and *Rhodanobacter*. The reshaping of the rhizosphere microbiome was accompanied by the upregulation of biological pathways related to metabolite biosynthesis in the rhizosphere. These modifications were related to the promotion of the growth and productivity of *P. heterophylla*. Our findings highlighted the significant role played by PGPR in medicinal plant yield and active ingredient accumulation when exposed to eCO_2_.

## 1 Introduction

Administration of plant growth-promoting rhizobacteria (PGPR) is an important and environmentally friendly method to achieve sustainable crop production (Bhattacharyya and Jha, 2012; Gray and Smith, 2005; Lugtenberg and Kamilova, 2009). The mechanisms associated with plant promotion commonly reported include direct promotion via the production of phytohormones and/or mobilization of nutrients in the soil, such as phosphorus (Liu et al., 2016; Richardson et al., 2009), and indirect promotion via inducing systemic resistance to biotic and/or abiotic stress (Akbar et al., 2022). While an increasing number of studies identified that biological amendments modified the composition and functionality of the rhizosphere microbial community, resulting in enhanced plant growth and health (Deng et al., 2022; Guo et al., 2024; Tao et al., 2023). The specific way in which PGPR affect soil microbiome varies depending on the PGPR strains, plant species, and environmental factors.

Recently, studies have revealed that the native rhizobacteria (Sun et al., 2022), fungi (Tao et al., 2023), and predatory protists (Guo et al., 2024) can be recruited of PGPR and cooperate as partners to enhance plant development (Sun et al., 2022; Zhang et al., 2023). These changes in indigenous organisms are indirectly influenced by elevated atmospheric CO_2_ (eCO_2_) (Kohler et al., 2009; Williams et al., 2018; Zytynska et al., 2020). Studies showed that the effects of eCO_2_ on microorganisms in the rhizosphere had large variability, and can be both positive (Jin et al., 2022) and negative (Li et al., 2022). Microbial growth, including bacteria, fungi, arbuscular mycorrhizal fungi, and actinomycetes can be stimulated under eCO_2_ (Lee and Kang, 2016). However, the high eCO_2_ may suppress the soil microbes and reduce their biomass (Xiao et al., 2017). These opposite effects happened through plant-mediated and soil-mediated mechanisms (Paterson et al., 1997; Montealegre et al., 2002). The phenotypic traits of PGPR related to plant growth, such as hormone indole-3-acetic acid secretion in *Pseudomonas* strains, are changed under 1,000 ppm conditions, contributing to establishing a developed root system that efficiently absorbs water nutrients from the soil (Sun et al., 2022; Tarnawski et al., 2006; Yu et al., 2016). The influenced root growth and rhizodeposition, in turn, potentially manipulate the indigenous microbial populations and activities (Redondo-Gómez et al., 2022; Sadowsky and Schortemeyer, 1997; Zak et al., 2000). The effects of eCO_2_ on microbes are also linked to the soil nutrient availability. For instance, under eCO_2_, reduced soil nitrogen availability resulting from enhanced plant nutrient uptake led to a decrease in microbial biomass (Butterly et al., 2016). Increased microbial biomass under eCO_2_ was associated with increased dissolved organic carbon in the soil (Hu et al., 2020). The functionality of recruited microorganisms is associated with competitive interactions (Wei et al., 2015) and secondary metabolism manipulation in the soil (Zhang et al., 2023). For example, bacteria strains with the ability to produce 6-hydroxypentadecanedioic acid (Cai et al., 2021) and antibiotics (Zhang et al., 2022), as well as the ability to increase peptide synthetase gene abundance (Deng et al., 2022) were enriched to protect against pathogen invasion. However, despite the understanding that PGPR and elevated CO_2_ are manipulations that can influence the composition and functioning of rhizosphere microbiomes for plant growth. It is unclear if these manipulations could enhance treatment efficacy or not when plants grow in a changing climate scenario. Regarding this, a better understanding of how additive PGPR impacts plant root development and indigenous soil microbiomes under eCO_2_ conditions is necessary to support more effective application strategies to improve host plant development.

The tuberous root of *Pseudostellaria heterophylla* (*P. heterophylla*) Rupr. & Maxim, also known as crown prince ginseng, has been a common traditional Chinese medicine for nearly 300 years. The below-ground tubers contain bioactive components, and tuber extractions are the most used materials for treating coronavirus disease 2019 in clinical practices (Alam et al., 2021; Ren et al., 2021; Wu et al., 2019). However, the tubers of *P. heterophylla* have a high incidence of *Fusarium* wilt infection, a disease caused by the soil pathogen *Fusarium oxysporum* (Chen et al., 2021; Liu et al., 2020). *Fusarium* wilt poses an ever-increasing threat to agriculture, causing significant declines in plant yields, such as banana, tomato, and medicinal plants, ranging from 20 to 90% annually (Strange and Scott, 2005; Tao et al., 2023; Yuan et al., 2022). As PGPR plays an essential role in pathogen control, they manipulate microbial resource competition networks (Wei et al., 2015), bacterial community diversity (Hu et al., 2016), and organism interactions (Huo et al., 2018). Although these studies revealed the importance of PGPR in pathogen suppression, how PGPR impacts the fungal genera *Fusarium* species presence under eCO_2_ and how these manipulations influence active ingredient accumulation in medicinal plants remains largely unknown.

The objectives of this study include (1) to investigate the differences in plant trait indicators; (2) to study the differences in soil microbiome composition and biological pathways; and (3) to reveal the relationship between plants and soil microbiome. *P. heterophylla* was grown in two separate chambers and inoculated with two types of PGPR strains (*Bacillus subtilis* and *Pseudomonas fluorescens*) under ambient CO_2_ and eCO_2_ conditions, respectively. The growth and productivity of *P. heterophylla* were monitored and evaluated by measuring its shoot, root, tuber yield, and active ingredient content. We first monitored the composition and diversity of the indigenous bacterial communities and the density of wilt pathogen *Fusarium* in the rhizosphere. Based on these results, we conducted the analysis to identify the changes in the key metabolites’ biosynthesis biological pathways related to the microbial community shift.

## 2 Materials and methods

### 2.1 Plant material and growth conditions

The experiments were conducted in two climate-controlled chambers in the Department of Civil and Environmental Engineering at The Hong Kong University of Science and Technology (HKUST) (22.3°N 114.2°E), from January to May 2022. The study used the *P. heterophylla* cultivar as the experiment plant material. Seed tubers were surface sterilized with 5% (v/v) sodium hypochlorite and washed four times with sterilized distilled water. The washed seed tubers were sown in a pot (8 cm diameter, 17 cm depth) filled with 1.7 kg soil collected from Bijie, Guizhou Province, China (Kong et al., 2021). Soil water condition (60% field capacity) was monitored with soil moisture probes and tensiometers (Ng et al., 2022a). The pots were watered every 2 days with deionized water. The basic physicochemical properties of soil are summarized in Table 1.

**Table 1:** Soil physicochemical properties after harvest.

Treatment	pH	EC (mS/cm)	C (μg/g)	N (μg/g)	P (μg/g)	IAA (μg/g)
aCK	5.47 ± 0.03d	1.43 ± 0.01c	198.27 ± 4.07b	166.55 ± 19.68a	175.34 ± 9.81b	197.34 ± 25.00c
aBS	5.81 ± 0.03a	1.14 ± 0.01e	208.00 ± 6.00b	113.48 ± 1.60c	140.40 ± 17.01c	283.35 ± 25.81b
aPF	5.83 ± 0.02a	1.01 ± 0.02f	220.38 ± 5.43a	164.22 ± 22.02ab	145.89 ± 25.95bc	349.39 ± 17.04a
eCK	5.67 ± 0.01c	1.49 ± 0.03b	220.26 ± 4.20a	143.08 ± 3.66b	242.39 ± 24.59a	223.17 ± 12.78c
eBS	5.49 ± 0.10d	1.59 ± 0.03a	207.10 ± 7.00b	171.34 ± 3.81a	214.12 ± 24.94ab	305.86 ± 14.67b
ePF	5.70 ± 0.02b	1.20 ± 0.02d	226.31 ± 3.81a	169.48 ± 10.22a	175.76 ± 19.66b	278.17 ± 13.42b

a: ambient CO_2_ condition (400 ppm). e: elevated CO_2_ condition (1,000 ppm). CK, control group without bacterial inoculation; BS, *Bacillus subtilis*-inoculated group; PF, Pseudomonas fluorescence-inoculated group; EC, electricity conductance. C: total organic carbon. N: total nitrogen. P: bioavailable phosphorus. IAA: indole-3-acetic acid. Data presented are the mean ± standard deviation (*n* = 3). Different letters beside the data indicate the significance of differences among treatments (*p* < 0.05).

The growth conditions are presented in Supplementary Figure S1. One of the chambers was set with an elevated CO_2_ concentration (eCO_2_), and the other was maintained at the average ambient CO_2_ concentration (aCO_2_). CO_2_ was supplied to the chamber via a compressed CO_2_ tank connected to a pipe within the chamber at the beginning of the experiments. Both chambers were set to a long-day photoperiod of 16 h:8 h, light: dark, at 23.5 ± 3°C. The light intensity was set to 79.198 mW/nm, and the relative humidity was maintained at 60 ± 10% (Chen et al., 2021).

### 2.2 Experiment treatments

The experiments were conducted in two independent chambers, one with eCO_2_ 1,000 ± 50 ppm (IPCC, 2022 predicted level) monitored and controlled using a remote sensor and an environmental controller (BETC-B2, Netherlands). The other one was with aCO_2_ concentration of approximately 400 ppm. In each chamber, plants were performed with 3 treatments: control groups (CK), a *B. subtilis*-inoculated group (BS), and a *P. fluorescens*-inoculated group (PF). All treatments were CK group grown under aCO_2_ (aCK) and eCO_2_ (eCK), BS group grown under aCO_2_ (aBS) and eCO_2_ (eBS), PF group grown under aCO_2_ (aPF) and eCO_2_ (ePF), respectively. To conduct the PGPR inoculation, the bacterial strains *B. subtilis* subsp. (GDMCC 1.372) and *P. fluorescens Migula* (GDMCC 1.782) were selected based on our previous study, and their beneficial traits were assessed (Ng et al., 2022b). The bacteria cultures were grown on nutrient agar for routine use and were maintained in Luria-Bertani broth with 15% glycerol at −80°C for long-term storage. The inoculated volume was 15 mL/pot of diluted PGPR solution at a concentration of 10^8 CFU/mL in the BS and PF groups during plant growth at 186-h intervals to ensure their colonization (Yadav et al., 2014). Due to the BS and PF inoculants being diluted with 0.9% NaCl solution, the same volume of 0.9% NaCl solution was inoculated into the CK group at the same frequency as the BS and PF groups. Each treatment contained 3 pots, and each pot contained 16 seed tubers at the beginning of the cultivation. During cultivation, the tuber production of *P. heterophylla* varied among individual plants. After harvest, the plants that exhibited tuber formation were counted into the replicates (12–19). Plants that did not form tubers and those that died due to reasons, such as germination failure and disease during the 150-day cultivation period were excluded from the count.

### 2.3 Plant growth, productivity, and disease incidence

To evaluate the growth changes in *P. heterophylla*, the shoot characteristics, including leaf area, number of leaves per plant, and shoot height, were monitored at five different time points after its germination: 60, 75, 90, 105, and 120 days. The leaf area was obtained with the software Image J (Abràmoff et al., 2004). Leaves numbers were counted and shoot height was measured with a ruler. Furthermore, we measured the photosynthetic parameters with the three fully expanded uppermost leaves of each plant. Stomatal conductance (g_s_) and soil plant analysis development (SPAD) were measured using a Leaf Porometer (SC-1, United States) and a chlorophyll meter (SLY-C, China), respectively. After 150 days of growth, plants were harvested and washed with deionized water (Supplementary Figure S2). Then, the dry weights of shoot, root, and tubers were recorded to calculate the ratio of shoot dry weight to height (W/H_shoot_), specific root length (SRL), tuber dry biomass, and harvest index (HI) based on Equations 1–4, respectively (Roberts, 2020; Bell and Fischer, 1994):


(1) $$\begin{array}{c} \mathrm{Ratio of shoot dry weight}\\ \mathrm{to height (g/cm)} \end{array}=\frac{\mathrm{Dry}\;\mathrm{weight of shoot}}{\mathrm{Shoot height}}$$

(2) $$\mathrm{Specific root length (cm/g)}=\frac{\mathrm{Root length}}{\mathrm{Dry}\;\mathrm{weight of root}}$$

(3) $$\begin{array}{c} \mathrm{Tuber}\;\mathrm{dry}\;\mathrm{biomass}\\ \left(\mathrm{mg}/\mathrm{plant}\right) \end{array}=\begin{array}{c} \mathrm{Dry}\;\mathrm{biomass of}\\ \mathrm{tubers}\;\mathrm{per}\;\mathrm{plant} \end{array}$$

(4) $$\mathrm{Harvest index}=\frac{\mathrm{Tuber}\;\mathrm{dry}\;\mathrm{biomass}}{\mathrm{Total}\;\mathrm{dry}\;\mathrm{biomass}\;\mathrm{per}\;\mathrm{plant}}$$

The maximum widths and lengths of the tubers were measured to present the morphology according to Hong Kong Chinese Materia Medica Standard (2020).

To assess the tuber quality, extraction and analysis of the active compounds comprised of polysaccharides (Hong Kong Chinese Materia Medica Standard, 2020), saponins (Ng et al., 2022a), and heterophyllin B (Zheng et al., 2019) were conducted. All tuber samples were dried at 65°C up to dryness via the oven-drying method and crushed to powder. To measure the polysaccharides, 0.5 g of the tuber powder was extracted with 30 mL of Milli-Q water in a water bath for 60 min. The polysaccharides content was measured using the anthrone-sulfuric acid colorimetric method at 625 nm with a UV–visible spectrophotometer (Lambda 950, Perkin Elmer, United States). To measure the saponins content, 0.1 g of dried powder was extracted with 40 mL methanol at 60°C for one hour. The saponin content was estimated using ginsenoside Rb1 as the reference standard. The absorbance of the solution was measured at 560 nm using a UV–visible spectrophotometer (Lambda 950, Perkin Elmer, United States). To measure the heterophyllin B content, 0.5 g of dried powder was ultrasonicated in 25 mL of ethanol for 45 min and filtered through a membrane (0.45 μm). high-performance liquid chromatography (HPLC) analysis was performed on a 1,200 series HPLC system (Agilent, United States). The one-point external standard method was used to calculate the content of heterophyllin B.

To calculate the disease incidence, the number of *P. heterophylla* tubers with wilt observation of disease symptoms, including wilting and dry brown rot, and the total number of *P. heterophylla* tubers after harvest in each treatment were counted. Each plant had one tuber, thus, the total number of tubers was 12–19 in all treatments. Disease calculation follows the below Equation 5.


(5) $$\begin{array}{l} \mathrm{Tuber disease incidence (\%)}=\\ \frac{\mathrm{Number of tubers with wilt observation}}{\mathrm{Number of total tubers}}\times 100\% \end{array}$$

### 2.4 Quantification of coupled effects

To assess the coupled effects of PGPR and eCO_2_, the independent action concept is used (Lasch et al., 2020). This method is applied to components with different modes of action, so the coupling effects can be calculated from the response of the individual components (Foucquier and Guedj, 2015). In the current study, the predicted effects’ values were calculated with Equation 6 (Alassane-Kpembi et al., 2017):


(6) $$\begin{array}{c} \mathrm{Coupled}\;\left(\mathrm{A+B}\right)\\ \mathrm{predicted value} \end{array}=\begin{array}{c} \left(\mathrm{mean value}\;\mathrm{A+mean value}\;B\right)-\\ \left(\mathrm{mean value}\;\mathrm{A\times mean value}\;B\right) \end{array}$$

A is the effects of PGPR, and B is the effects of eCO_2_. The combination index (CI) is used to distinguish the different effects. CI is defined in Equation 7:


(7) $$\mathrm{CI=}\frac{\mathrm{Coupled}\;\left(\mathrm{A+B}\right)\;\mathrm{predicted value}}{\mathrm{Coupled}\;\left(\mathrm{A+B}\right)\mathrm{measured value}}$$

The empirical threshold of CI < 0.9 is set as the threshold indicates synergistic effects (synergism in PGPR and eCO_2_). 0.9 ≤ CI ≤ 1.1 is set to indicate the additive effects, and CI > 1.1 is set to indicate antagonistic effects (Chou, 2006).

### 2.5 Soil sampling, DNA extraction, and metagenome sequence analysis

To collect the rhizosphere soil, *P. heterophylla* plants were carefully uprooted from each pot. The rhizosphere soil was obtained by gently shaking off loosely attached soil around the roots and collecting the soil tightly attached to the roots (Akbar et al., 2022). Subsequently, the collected soil samples were then homogenized by pot. Each treatment had three pots, and therefore, the rhizosphere soil sample had three replicates per treatment. The soil samples were sieved through a 2-mm mesh, and a portion of each sample was stored at −80°C for total DNA extraction. The remaining soil was air-dried and used for nutrient and indole-3-acetic-acid (IAA) determination following Ng et al. (2022b) and Yan et al. (2022). The total DNA extraction was performed using an E.Z.N.A. Soil DNA Kit (Omega Bio-tek, Norcross, GA, United States). The quality of extracted soil DNA was assessed with a NanoDrop 2000 Spectrophotometer (Thermo Scientific, Waltham, MA, United States).

Metagenome sequence analysis was conducted at the Beijing Genomics Institute (BGI, Hong Kong, China). The DNA from each sample was fragmented, end-repaired, and adenylated. Adaptors were then ligated to the ends of the fragments, and a polymerase chain reaction (PCR) was carried out to amplify the product. The PCR product was then subjected to circularization, and single-stranded circular DNA molecules were produced via rolling cycle amplification. These were loaded onto patterned nanoarrays and sequenced using combinatorial Probe-Anchor Synthesis. All the raw data were trimmed using SOAPnuke v.1.5.2, and the trimmed reads were mapped to the host genome to remove host-originated reads (only for samples of host origin). High-quality reads were assembled using MEGAHIT software, and contigs with lengths less than 200 bp were discarded. Genes were predicted using MetaGeneMark, and redundant genes were removed using CD-HIT with identity and coverage cutoffs of 95 and 90%, respectively. Salmon software was used for quantification. Moreover, annotation information was generated by aligning the protein sequences of genes against a functional database (like KEGG) using DIAMOND. Taxonomic annotation was assigned based on the Kraken LCA algorithm, and Bracken software was used to generate the taxonomic and functional abundance profiles. Wilcoxon’s rank sum test was used to determine the features with significantly differential abundances across groups, and differentially enriched KEGG pathways were identified using reporter scores. Statistical analysis of the Wilcoxon rank test and the Kruskal-Wallis H test were performed using the R project.

### 2.6 Statistical analysis

The normality of the soil physicochemical properties data and plant-associated data was analyzed and represented as the mean with standard deviation. Statistical analysis was conducted using the software package SPSS version 20.0. Differences between mean values of soil physicochemical properties and plant-associated parameters were assessed via a two-way analysis of variance (ANOVA) followed by *post hoc* comparisons using the least significant difference (LSD) test (Yang et al., 2022). Significance was assessed at the 95% confidence interval (*p* < 0.05). Microbial composition and diversity results were analyzed in the RStudio (Version 2023.06.1). The alpha diversity was quantified by the Shannon, Chao1, and Simpson indices using the relative abundance profiles at the species level with the R package “vegan” (Oksanen et al., 2019). The beta diversity was presented with principal coordinate analysis (PCoA) based on the Bray-Curtis distance. The permutational multivariate analysis of variance, function adonis, transformed data by Bray-Curtis, permutation = 999 (PERMANOVA) was used to analyze the differences in the microbial community profiles with the “vegan” package (Zhang et al., 2023). Mantel test was performed using the “vegan” package in R to determine correlations between plant–soil factors and rhizosphere microbial communities. Spearman’s correlation analysis of dominant bacterial genera and biological pathways in the rhizosphere was performed using the “psych” package in R (Zhang et al., 2023). Random Forest analysis was used to disentangle the potential main predictors of *Fusarium* pathogen, harvest index, and active compound contents (Ding et al., 2022).

## 3 Results

### 3.1 Plant photosynthesis performance, shoot, and root development

**Figure 1:**
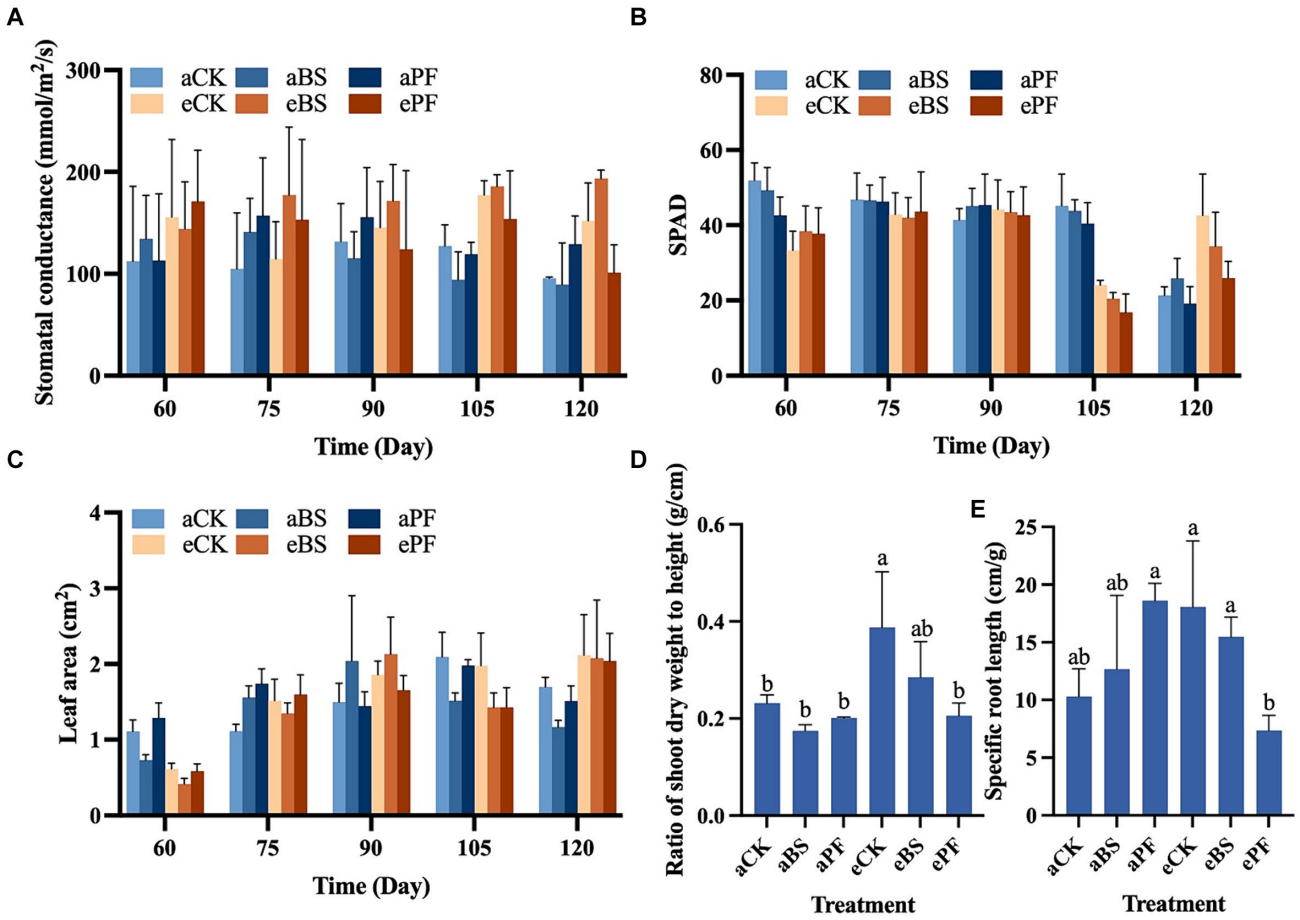
Growth traits for *P. heterophylla* individuals cultivated with inoculation of PGPR (*B. subtilis* and *P. fluorescens*) under different atmospheric CO_2_ levels (ambien: 400 ppm, elevated: 1000 ppm) at 60, 75, 90, 105, and 120 days of development. **(A)** Stomatal conductance. **(B)** Soil plant analysis development (SPAD). **(C)** Leaf area. **(D)** Ratio of shoot dry weight to height. **(E)** Specific root length. Bars represent the mean with standard deviation (*n* = 12–19). Different letters on the top of each bar indicate the significance of differences among treatments (*p* < 0.05).

The photosynthesis performance of *P. heterophylla* was significantly affected by eCO_2_ treatment and eCO_2_ coupled with BS treatment. With the elevation of CO_2_, the stomatal conductance (g_s_) showed a pronounced increase in treatments at eCO_2_ conditions, especially at 105 and 120 days (Figure 1A). The g_s_ describes the rate of gas exchange (e.g., CO_2_ uptake) and functions as the measure of stomatal opening in response to environmental conditions. Greater g_s_ indicates the photosynthesis rate is higher. Although inoculating PGPR also increased the g_s_ than control treatments, the differences were not significant. Higher g_s_ values were observed in the eBS treatments from 75 days to 120 days. Soil plant analysis development (SPAD) measures leaf chlorophyll concentrations and larger values indicating higher leaf photochemical efficiency. The eCK and eBS treatments at 120 days exhibited larger SPAD values than aCK (Figure 1B). Shoot development including leaf area, leaf number, and shoot height was increased by PGPR and/or eCO_2_ (Figure 1C; Supplementary Figure S3). The leaf area showed a remarkable increase from 60 to 75 days of plant development. It increased by 20–25% at 120 days in eCK, eBS, and ePF treatments, thereby surpassing other treatments during this period (Figure 1C). PGPR coupled with eCO_2_ enhanced the shoot development, with higher shoot height and larger ratio of shoot dry weight to height (W/H_shoot_) were observed at 75 and 105 days (Figure 1D). Compared to aCK, W/H_shoot_ increased by 70 and 26% at eCK (*p* < 0.05) and eBS, respectively. The root development, especially the specific root length (SRL), was improved under eCO_2_ (Figure 1E).

### 3.2 Plant productivity and disease incidence

**Figure 2:**
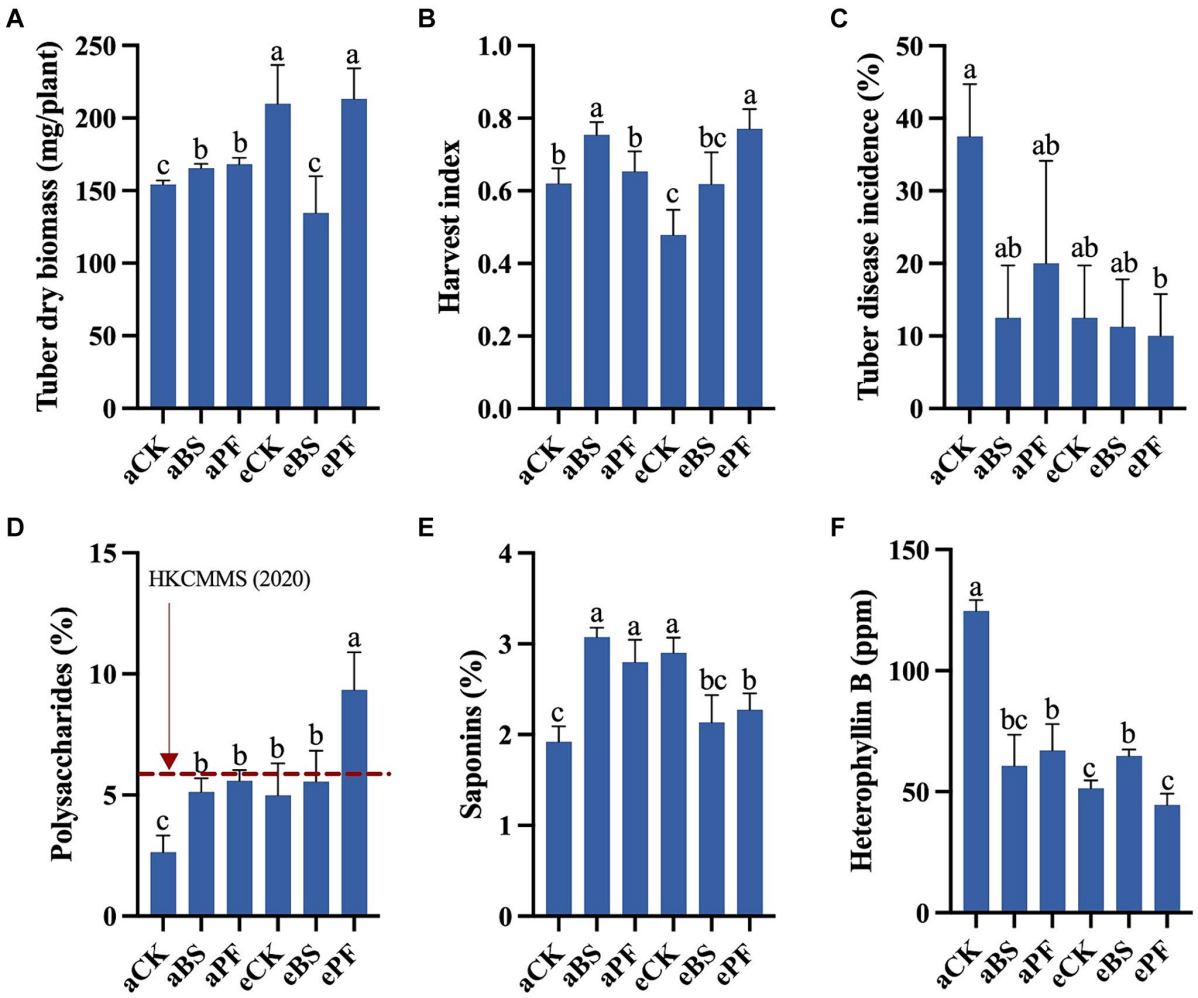
Tuber yield, quality, and disease incidence of *P. heterophylla* with different treatments. **(A)** Tuber dry biomass. **(B)** Harvest index. **(C)** Tuber disease incidence. **(D)** Proportion of polysaccharides in tubers. **(E)** Proportion of saponins in tubers. **(F)** Proportion of heterophyllin B in tubers. Bars represent the mean with standard deviation (*n* = 12–19). Different letters on the top of each bar indicate the significance of differences among treatments (*p* < 0.05).

The productivity of *P. heterophylla* was described by yield (tuber biomass and harvest index) and tuber quality (the contents of the active ingredients) (Figure 2). Compared to aCK, the tuber dry biomass significantly increased by 7, 36, and 38% in aBS, eCK, and ePF, respectively. Additionally, the harvest index showed enhancements ranging from 5 to 24% in PGPR and eCO_2_-treated groups. The ePF had plants with the largest tuber dry biomass at 213 mg/plant, as well as the highest harvest index at 0.77 (Figures 2A,B). Taken together, the results showed that the yield of *P. heterophylla* was enhanced the most in the ePF treatment.

Compared to aCK, the proportions of polysaccharides were significantly increased from 2.65 to 9.35%, with a 253% increase (Figure 2D). The highest polysaccharide content was found in ePF, at 9.3% (g/g), which is over 5 times higher than the content found in tubers grown in Anhui Province (1.62%, g/g) (Deng et al., 2018). The percentages of saponins were significantly increased by 60, 46, 51, and 19% in aBS, aPF, eCK, and ePF, respectively, compared to aCK (Figure 2E). The aBS showed the highest saponins content of 3.1% (g/g), which is nearly 7 times higher than the content found in tubers grown in Fujian Province (Ma et al., 2018). As for the heterophyllin B, applying PGPR and eCO_2_ decreases its percentages by 46–64% relative to aCK (Figure 2F). Overall, the polysaccharides and saponins proportions were positively increased with PGPR and eCO_2_, while heterophyllin B proportion in tubers was negatively decreased.

**Figure 3:**
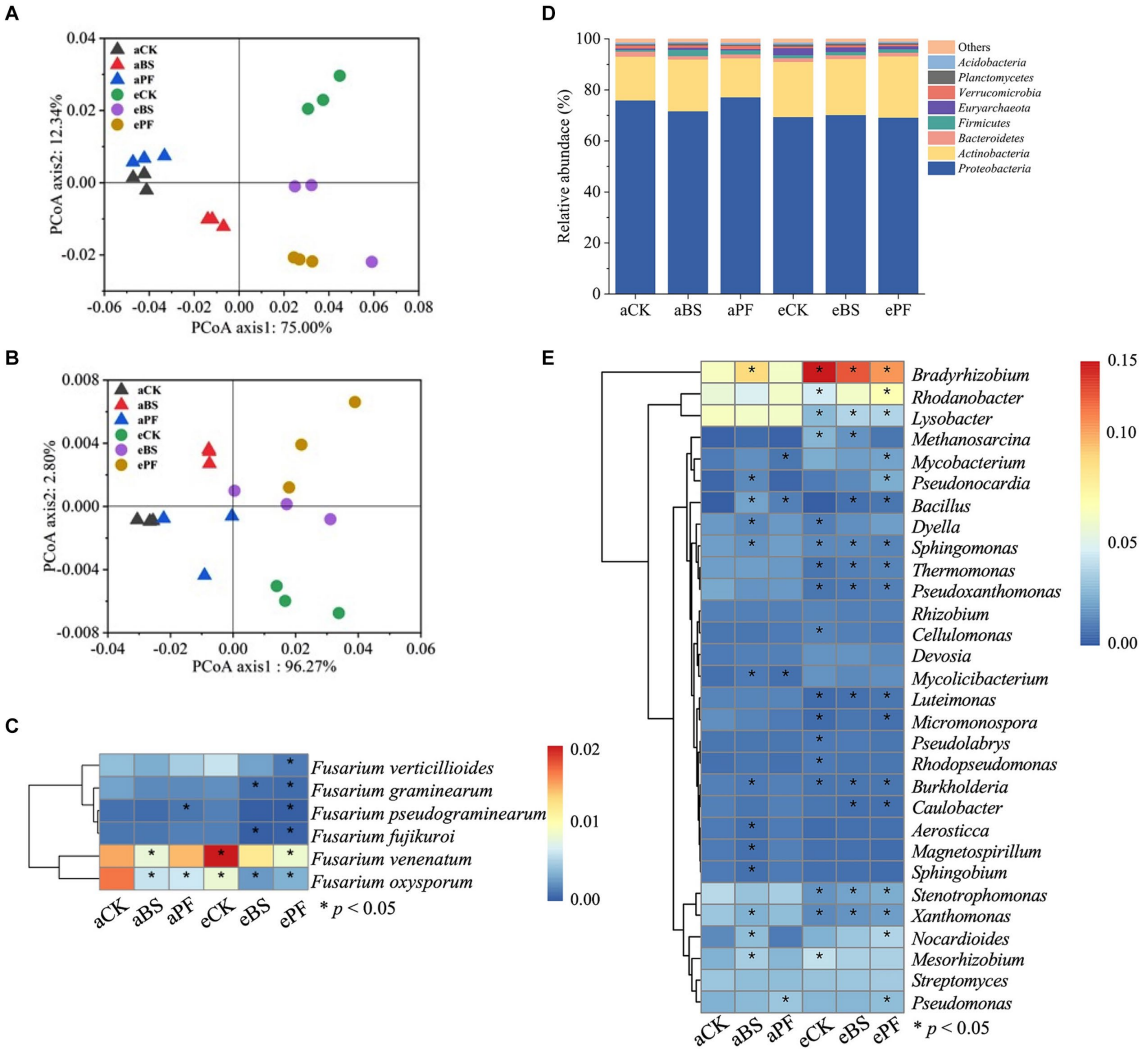
The structure and composition of soil microbiome. PCoA was used to visualize the dissimilarity of **(A)** bacterial and **(B)** fungal compositions. **(C)** Relative abundance of fungus *Fusarium*. Relative abundance of bacterial communities at the **(D)** genus level and **(E)** phylum level. The asterisks in the boxes of the heatmaps **(C,E)** indicate the significance of differences among treatments (*p* < 0.05).

Inoculating with PGPR and/or elevation of CO_2_ reduced tuber disease incidence by 46.7 to 73.3% compared to the control group (Figure 2C). Although the PGPR or eCO_2_ resulted in a lower tuber disease incidence than aCK, the difference was not significant. The most significant decrease in tuber disease incidence was found in the ePF treatment with a significant 73.3% reduction (*p* < 0.05). The relative abundance of *F. oxysporum* in the rhizosphere showed similar trends as the disease incidence (Figure 3).

### 3.3 Structure and composition of the indigenous microbial community

PCoA results of the beta diversity showed that the untreated group, i.e., aCK, was clearly separated from other treatments (Figure 3A), suggesting PGPR and eCO_2_ influenced the indigenous bacterial community, but the fungal community change was less evident (Figure 3B). Similar to the Chao 1 index, the elevation of CO_2_ only increased the alpha diversity of bacterial community (Supplementary Table S2). The fungus *Fusarium* associated with wilt disease showed a marginal decrease in density in response to PGPR and eCO_2_ application. As depicted in Figure 3C, compared with aCK, the relative abundance of *F. oxysporum* significantly decreased by 57, 57, and 45% in aBS, aPF, and eCK, respectively. Moreover, its abundance was further decreased in eBS and ePF, with a 78 and 72% reduction being observed instead, relative to aCK.

Within the composition of bacterial communities, at the phylum level (Figure 3D), *Proteobacteria* and *Actinobacteria* were the most dominant across all treatments. Compared to aCK, the application of PGPR and eCO_2_ significantly increased the relative abundance of *Actinobacteria* by 18–40%, except for the aPF treatment. The dominant bacterial genera were also altered by applying PGPR and eCO_2_ (Figure 3E). The relative abundances of *Bradyrhizobium*, *Rhodanobacter*, and *Mesorhizobium* were significantly higher in the PGPR coupling with eCO_2_ treatments. The relative abundances of *Lysobacter* and *Stenotrophomonas* were significantly lower in the PGPR coupling with eCO_2_ treatments.

### 3.4 Changes in metabolites biosynthesis biological pathways in indigenous microbiome

**Figure 4:**
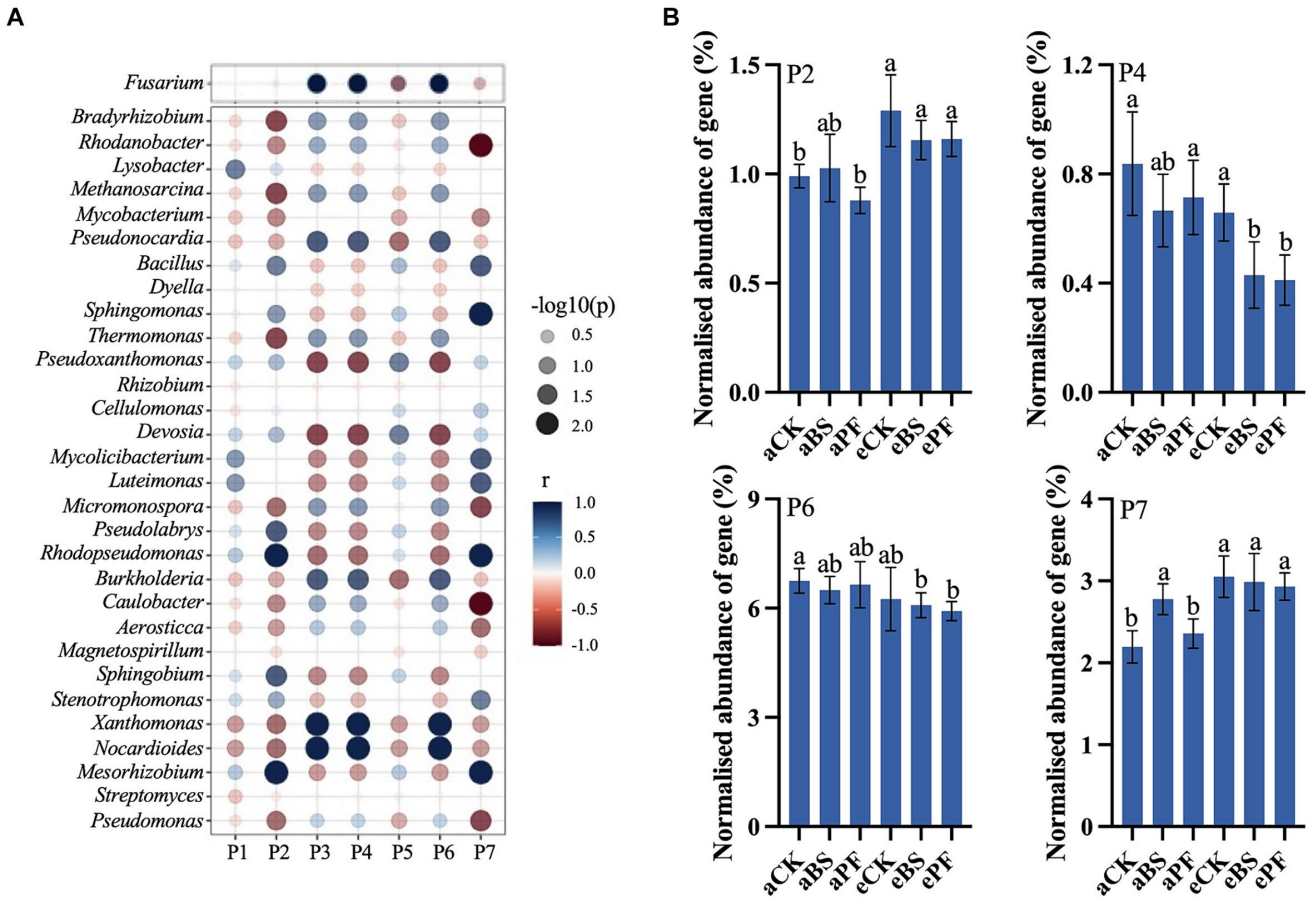
**(A)** Spearman correlation between soil microbes and biological pathways. **(B)** KEGG analysis of the normalized abundance of genes in response to PGPR and eCO_2_. P2: Biosynthesis of various alkaloids. P4: Biosynthesis of various other secondary metabolites. P6: Plant-pathogen interaction. P7: Tetracycline biosynthesis. Bars in **(B)** represent the mean with standard deviation (*n* = 3). Different letters on the top of each bar indicate the significance of differences among treatments (*p* < 0.05).

KEGG biological pathway analysis revealed major transcriptional alterations in different pathways (Figure 4). Spearman correlation analysis showed that soil microbial components were correlated to biological pathways prominence in the rhizosphere (Figure 4A; Supplementary Figure S6). The fungus *Fusarium* and dominant bacterial genera were significantly related to antibiotics biosynthesis pathways and secondary metabolisms pathways (*p* < 0.01). The application of PGPR and eCO_2_ upregulated the pathways associated with antibiotic biosynthesis, including tetracycline, clavulanic acid, macrolides, and various alkaloids. The normalized abundance of genes in the tetracycline biosynthesis and biosynthesis of various alkaloids were significantly increased by 17–36% in eBS and ePF, compared to aCK (Figure 4B). In contrast to antibiotic biosynthesis, the plant-pathogen interaction and biosynthesis of various other secondary metabolite pathways were downregulated, suggesting relieved pathogen stress and decreased production of some phytohormones.

### 3.5 Relationship between plant and indigenous microbiome

**Figure 5:**
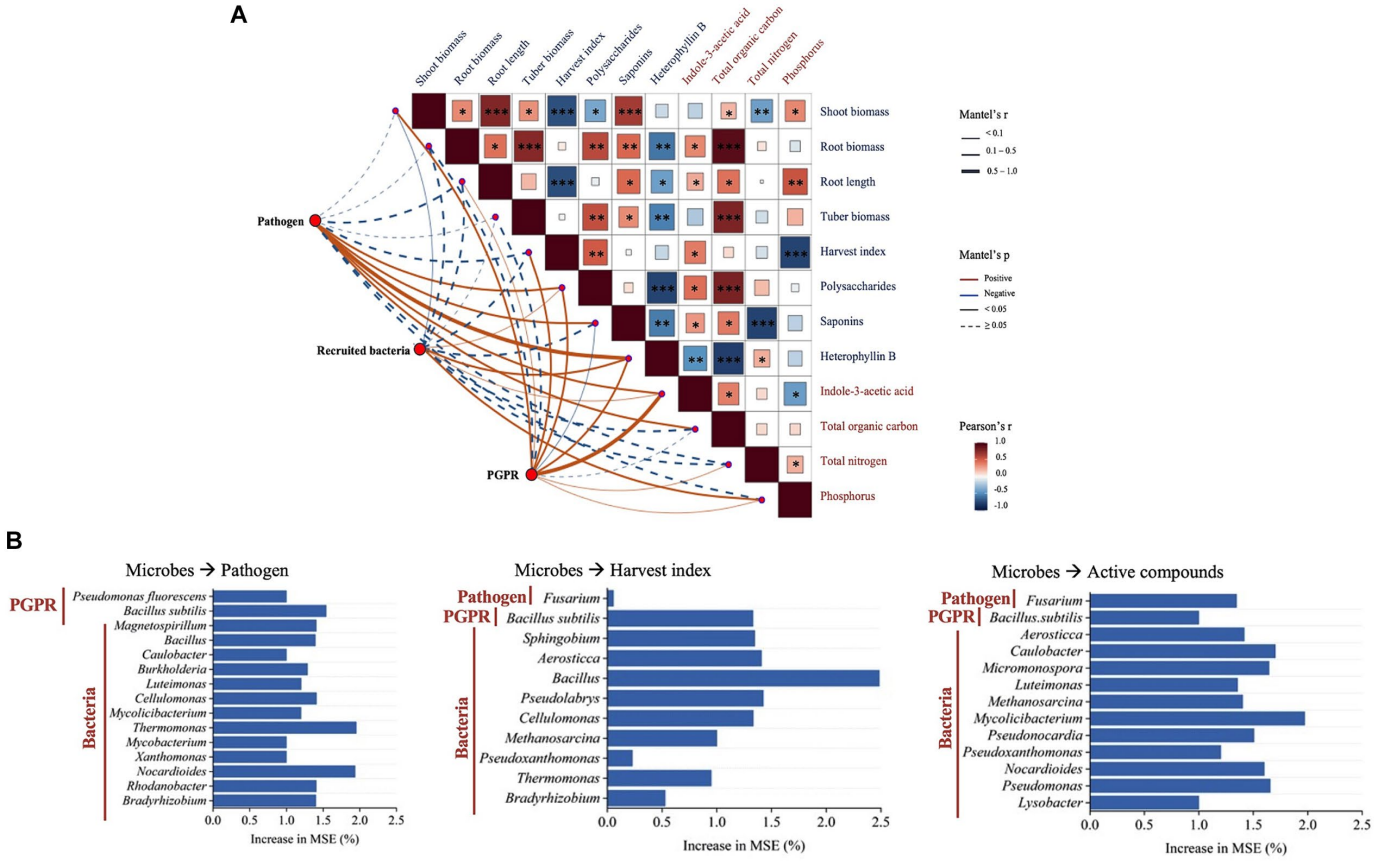
**(A)** Mantel test of the relationship between plant characteristics, rhizosphere microbial community components, and soil properties. The asterisks in the box *, **, and *** represent significant differences at the 0.05, 0.01, and 0.001 levels, respectively. **(B)** Random Forest analysis of predictors importance [percentage of increase in mean square error (MSE)] of rhizosphere microbes as drivers for the *Fusarium* pathogen, harvest index, and active compound contents in the tubers of *P. heterophylla*. Higher MSE% values imply more important predictors.

The Mantel test results showed the relationships in the plant–soil network. Plant characteristics were connected to soil microbial components and soil physicochemical properties (Figure 5A). The abundance of *Fusarium* pathogen was negatively related to the harvest index and root development. The abundance of inoculated PGPR was positively related to rhizosphere IAA. Pearson’s correlation analyses showed that rhizosphere IAA was a crucial factor affecting root development, harvest index and the proportions of active compounds. Total organic carbon was positively related to root biomass, tuber biomass, and the content of polysaccharides. Additionally, soil bioavailable phosphorus was also related to root growth.

The Random Forest model results showed that rhizosphere microbial components were important predictors for the *Fusarium* pathogen presence, tuber harvest index, and active compound contents (Figure 5B). Two types of PGPR and thirteen recruited bacteria genera as the main microbial predictors of the *Fusarium* pathogen abundance in the rhizosphere. Among nine bacteria genera selected for predicting harvest index, *Bacillus* was the most significant indicator. Regarding the active compound contents, *Fusarium* and eleven bacteria genera were identified as potential microbial predictors.

Pearson correlation analysis revealed that the tuber yield was positively related to root length in all treatments except for aBS and eBS (Supplementary Figure S7). Instead, strong positive correlations among the photosynthetic parameters and tuber yield were found in aBS and eBS treatments. In aCK treatments, tuber quality was positively correlated to the leaf area, W/H_shoot_, and root development. A positive relation was found between the content of polysaccharides and root development in treatments at aCO_2_ conditions. While polysaccharides contents showed a positive correlation with leaf area in eBS and ePF treatments.

## 4 Discussion

### 4.1 Coupled effects of PGPR and eCO_2_ on plant growth and productivity

To quantify the coupled effects of two environmental manipulations (PGPR and eCO_2_) on plant growth and productivity, we used the independent action concept by calculating the combination index (CI) (Table 2). Empirical thresholds of the CI < 0.9, 0.9 ≤ CI ≤ 1.1, and CI > 1.1 are set to indicate synergistic effects (synergism), additive effects, and antagonistic effects (antagonism), respectively (Chou, 2006; Lasch et al., 2020). Applying PGPR and eCO_2_ together showed an additive effect (CI: 0.98) in the increased tuber yield (dry biomass and harvest index) (Table 2), with the dry biomass of tubers in this study approximately 1.5 times higher than that of a previous study (Ng et al., 2022a). This can be attributed to the promoted root development in ePF, as a strong positive correlation was found between tuber yield and root development (Figure 5; Supplementary Figure S7). The ratio of root length to dry weight (specific root length, SRL) provides a ratio of a standard unit of acquisition (root length) to resource investment (biomass) (Kramer-Walter et al., 2016; Poorter and Ryser, 2015). It characterizes economic aspects of the root system and is indicative of environmental changes, such as elevated CO_2_ concentrations (Ostonen et al., 2007). In Figure 1E, the results showed the SRL of *P. heterophylla* increased slightly under eCO_2_, suggesting enhanced nutrient uptake. This observation was different from Ostonen et al. (2017) reported that there was a decrease in the SRL of trees. The reason may be that root development varies from species to species, and it relies on growth conditions, root order, and root locations (Crookshanks et al., 1998). Moreover, PGPR inoculation significantly increased IAA in the rhizosphere, which was significantly correlated with average root length (*p* < 0.01) (Supplementary Figure S4). IAA induces vascular differentiation in the root system and enhances root development, contributing to nutrient and water uptake for plant vigor (Tarnawski et al., 2006). This finding was consistent with the results of another bacteria strain, *P. stutzeri* XL272, which stimulated cucumber growth through IAA production (Sun et al., 2022). The harvest index illustrates biomass allocation. A higher harvest index in aBS and ePF indicated more efficient resource allocation to the economically valuable part (tuber), potentially increasing profitability. The increased harvest index can be attributed to the improvement in shoot and root development with the application of PGPR and/or eCO_2_. Higher allocation of resources to root biomass may result in reduced resource allocation to above-ground shoots (Poorter and Ryser, 2015). Changes in the ratio of shoot dry weight to height (W/H_shoot_) describe the response of plant morphology under different growth conditions. For example, *P. heterophylla* showed a larger W/H_shoot_ under higher CO_2_ conditions (Figure 1D), suggesting elevated CO_2_ improved plants lateral growth more than vertical growth (Wu et al., 2004).

**Table 2:** Summary of combination index of coupled effects of PGPR and eCO_2_.

		Combination index	
		*B. subtilis* + eCO_2_	*P. fluorescens* + eCO_2_
Tuber morphology	Maximum width	1.24 (ant.)	1.05 (add.)
	Length	1.43 (ant.)	1.65 (ant.)
Yield	Tuber dry biomass	1.55 (ant.)	0.98 (add.)
	Harvest index	1.24 (ant.)	0.94 (add.)
Active compound contents	Polysaccharides	1.33 (ant.)	0.81 (syn.)
	Saponins	1.44 (ant.)	1.34 (ant.)
	Heterophyllin B	1.34 (ant.)	2.04 (ant.)

eCO_2_, elevated CO_2_ condition (1,000 ppm); CI, combination index. CI < 0.9, 0.9 ≤ CI ≤ 1.1, and CI > 1.1 were set to indicate synergistic effects (syn.), additive effects (add.), and antagonistic effects (ant.), respectively (Chou, 2006).

The content of active compounds in medicinal crops is crucial for their curative properties (Wei et al., 2020). However, little attention has been given to understanding how these qualities are affected by growth conditions and microorganisms. This study revealed that the proportion of three active compounds: polysaccharides, saponins, and heterophyllin B, were significantly influenced (Table 2). The synergistic promotion (CI: 0.81) of polysaccharides content in ePF was significant. The promotion may be due to the increased leaf area (Figure 1C), leading to higher photosynthesis and more carbohydrate accumulation in tubers (Burlak et al., 2013). Additionally, root dry weight and average root length showed increases in PGPR and eCO_2_-treated groups (Supplementary Figure S3), which may enhance transpiration and partially mitigate the negative effects of eCO_2_. The transpiration process drives increased carbohydrate transport to the tuber (Sun et al., 2022).

In contrast to polysaccharides, an antagonistic effect (CI: 1.1) of PGPR coupling with eCO_2_ on the increased saponins and heterophyllin B over aCK was found. Their contents were negatively correlated with total nitrogen and total organic carbon in the soil and were affected by microbial components. This observation was in agreement with a previous study conducted by Xu et al. (2024) that identified the significant role of microbial communities in regulating soil nutrients. Developing a denser network of root hairs and lateral roots in eBS and ePF (Section 3.1) helped improve nutrient absorption ability. However, the higher uptake of microelements, such as Cu and Mn, negatively affected saponin biosynthesis (Wei et al., 2020; Ng et al., 2022a). Heterophyllin B is a cyclic octapeptide whose formation needs nitrogen-contained precursors. The downregulation of the biological pathways, like the biosynthesis of amino acids and various secondary metabolites, may influence its formation (Zheng et al., 2019; Ruan et al., 2023).

Tuberization is a complex process involving interaction between various genetic, biochemical, and environmental factors (Singh et al., 2015). Plants without tuber formation may be due to the varied temperatures during growth, which is one of the most significant factors that affect tuber formation. Besides, the condition factors, such as photoperiod, light (intensity and quality), mineral nutrition, water availability and pathogens can influence tuberization (Abelenda et al., 2014). In addition, at the beginning of the cultivation, some of the seed tubers failed to germinate, which may lead to an increased death rate. Moreover, the survival rate of plants excluding disease ranged from 58 to 75% (Supplementary Figure S9), which probably implied that a high rate of plants died because of disease. Additionally, PGPR inoculants significantly increased the maximum width of tubers under aCO_2_ conditions (*p* < 0.05) (Supplementary Table S1), suggesting the tuber shape changed from elongated to ellipsoid. This may be due to the changes in the ATP/ADP translocator, which is the only protein dominant in the tuber morphology (Fernie and Willmitzer, 2001).

### 4.2 Suppression of fusarium pathogen by biocontrol bacteria in the rhizosphere

In this study, aCK had the highest abundance of the pathogen fungus *Fusarium*, which has a strong predictive importance for wilt incidence and crop quality (the content of nutrients and active ingredients) reduction (Guo et al., 2022; Tao et al., 2023). As a result, the tubers in aCK show wilting and dry brown rot, and its polysaccharides and saponins contents were lower than tubers of other treatments (Supplementary Figure S5). However, after applying PGPR and eCO_2_, the *Fusarium* density, in particular *F. oxysporum*, significantly decreased. It has been demonstrated that reductions of *Fusarium* indicated a relieved pathogen threat to plants, leading to a decrease in wilt disease incidence (Deng et al., 2022). The decrease in disease incidences and pathogen densities is associated with the PGPR inoculation and elevation of CO_2_. The disease-suppressive function of PGPR and eCO_2_ is related to interactions between pathogenic fungus *F. oxysporum* and bacteria. Previous studies showed that the abundance of dominant bacteria families potentially contributed to the pathogen abundance in the rhizosphere of tomato plants (Deng et al., 2022). Our results enforce the suppression potential of the bacterial communities recruited in soils. They also complement previous findings indicating that bacterial functions related to metabolite biosynthesis pathways can effectively increase plant health and quality by reducing pathogenic fungi in various plant-pathogen systems (Guo et al., 2024; Wei et al., 2015). This suppressed growth of *Fusarium* is in line with research that showed the inhibitory effects of probiotic agents on *F. oxysporum* growth in other herbal plants, e.g., American ginseng (Boulahouat et al., 2023; Dukare et al., 2021; Liu et al., 2020) and tomato plants (Guo et al., 2024). Different from previous research, the decrease in *Fusarium* density was higher under eCO_2_ conditions. Previous studies revealed the possible mechanism of PGPR against disease pathogens was reshaping the indigenous microbiome composition and function (Deng et al., 2022; Wu et al., 2021). Specifically, the beneficial strains, mostly *Pseudomonas poae*, were recruited into the rhizosphere of *P. heterophylla* to resist *Fusarium* wilt (Yuan et al., 2022). However, our study found no significant variance in the relative abundance of *Pseudomonas poae* among all treatments (Supplementary Figure S9). It is instead identified that the relative abundance of the dominant phylum *Actinobacteria* and the genera, e.g., *Rhodanobacter*, *Bradyrhizobium*, and *Mesorhizobium*, is increased in response to PGPR and eCO_2_ treatments (Figure 3). As reported by Huo et al. (2018) and Palaniyandi et al. (2013), *Actinobacteria* and *Rhodanobacter* are prolific antibiotic producers in soil and can produce functioning metabolites, like antibiotics, to suppress pathogen growth. As such, it can be said that applying PGPR at eCO_2_ recruits indigenous biocontrol bacteria, which can contribute to the suppression of *Fusarium* pathogens.

### 4.3 Regulation of metabolites biosynthesis biological pathway to reshape microbial community

The role of PGPR coupling with eCO_2_ in rhizosphere microbial communities has been investigated using microbial analysis (Yu et al., 2016). To further examine the biocontrol mechanisms at the molecular level, this study takes advantage of metagenomic analyses to characterize the transcriptional outcomes of microbe interactions in the soil. It was found that distinct gene expression profiles were present in the PGPR and eCO_2_ treatments compared to the control. The biological pathways related to functioning metabolite biosynthesis significantly correlated with the abundance of *Fusarium* and indigenous dominant bacterial genera (Figure 4). This agrees with studies conducted by Sun et al. (2022) and Deng et al. (2022), which concluded that metabolic interactions play a significant role in reshaping the rhizosphere microbiome. With KEGG analysis, previous studies identified microbial agents against *Fusarium* disease in watermelons and tomatoes by manipulating metabolic pathways related to the synthesis of lignin, acid, and antibiotics (Cai et al., 2021; Zhang et al., 2022). Consistently, this study found that four typical pathways of antibiotic biosynthesis (tetracycline, alkaloids, macrolides, and clavulanic acid) were upregulated, two of which were significant, namely tetracycline and alkaloids. Rhizobacteria *Actinobacteria* has evolved an excellent ability to produce tetracycline, and *Pseudomonas* can produce alkaloids, which has been demonstrated by previous research (Klapper et al., 2016; Lozano et al., 2019; van der Heul et al., 2018). Applying PGPR coupled with eCO_2_ significantly increased the abundance of *Actinobacteria* and *Pseudomonas*, strengthening our findings that PGPR coupled with eCO_2_ contributes to the biosynthesis of antibiotics by recruiting biocontrol bacteria. Thus, *Fusarium* pathogen growth can be suppressed by the above biocontrol bacteria via upregulating antibiotic biosynthesis in the PGPR and eCO_2_ treatments. This was further demonstrated by the decreased abundance of *Fusarium* in Figure 3. Akbar et al. (2022) reported that the plant-pathogen interaction is a significant player in mediating the plant immune response against invading pathogens. The relieved pathogen stress led to a downregulated plant-pathogen interaction, suggesting a decreased pathogen infection in plants following the application of PGPR coupled with eCO_2_. By creating a favorable and healthy rhizosphere, the growth and productivity of *P. heterophylla*, including yield and quality, in PGPR and eCO_2_-treated groups were enhanced more effectively.

The structure and composition of rhizosphere microbial communities are crucial for plant growth and health (Wei et al., 2019). The individual effects of PGPR or eCO_2_ on microbial communities have already been well-studied (Gray and Smith, 2005; Kohler et al., 2009; Šibanc et al., 2014; Yilmaz and Karik, 2022). However, the response patterns of microbial communities to interactions between different manipulations (e.g., CO_2_ elevation and PGPR inoculation) remain largely unknown. *P. fluorescens* coupled with eCO_2_ slightly decreased the alpha index, reducing species richness and evenness (Supplementary Table S2). These changes were not observed when PGPR or eCO_2_ treatments were administered separately, in which bacterial diversity was either unaffected or increased (Berg et al., 2020; Finkel et al., 2020; Jia et al., 2023). This might be due to plants in soil inoculated with PGPR at eCO_2_ favoring certain bacterial species over others, as observed in Figure 3. This favor was also found in other plant species, such as barley (Zytynska et al., 2020), *Leymus chinensis* (Li et al., 2022), and *Brassica napus* (Mamet et al., 2022). Bacteria that best contributed to plant growth in the rhizosphere were greatly favored in this environment and, as such, were grown in greater abundances, leading to lower bacterial diversity. To bridge links between plant physiology and microbial activities under eCO_2_, molecular signaling evaluations, including the role of osmolytes and reactive oxygen species, should also be explored. Additionally, further study should incorporate data on plant physiological parameters that would provide valuable insights into the mechanisms of plant-microbe interactions.

## 5 Conclusion

In summary, this study evaluated the coupled effects of different environmental manipulations on plant growth and productivity as well as the rhizosphere microbiome within a medicinal plant cultivation system. We provided evidence that increased root development and indigenous beneficial bacteria were associated with plant productivity (yield and active ingredient accumulation) in response to PGPR under eCO_2_. Specifically, the recruitment of biocontrol bacteria probably is effective in inhibiting the growth of the pathogenic fungus *Fusarium*. The increase in these probiotics was accompanied by the upregulation of biological pathways related to the biosynthesis of secondary metabolites, which may contribute to the suppression of *Fusarium*. These findings highlighted the importance of PGPR under eCO_2_ in improving active ingredient accumulation in medicinal plants. The balanced interaction between rhizosphere probiotics and *Fusarium* pathogen could be facilitated by the combined environmental manipulations, demonstrating the potential for sustainable agriculture practices. However, further studies on *in vitro* investigations of *Fusarium* and field applications with long-term cultivation are still needed.

## Data Availability

The datasets presented in this study can be found in online repositories. The names of the repository/repositories and accession number(s) can be found at: https://www.ncbi.nlm.nih.gov/, PRJNA1083094.

## References

[ref1] Abelenda J. A. Navarro C. Prat S. (2014). Flowering and tuberization: a tale of two nightshades. Trends Plant Sci. 19, 115–122. doi: 10.1016/j.tplants.2013.09.010, PMID: 24139978

[ref2] Abràmoff M. D. Magalhães P. J. Ram S. J. (2004). Image processing with ImageJ. Biophoton. Int. 11, 36–42. Available at: https://imagescience.org/meijering/publications/download/bio2004.pdf

[ref3] Akbar A. Han B. Khan A. H. Feng C. Ullah A. Khan A. S.. (2022). A transcriptomic study reveals salt stress alleviation in cotton plants upon salt tolerant PGPR inoculation. Environ. Exp. Bot. 200:104928. doi: 10.1016/j.envexpbot.2022.104928

[ref4] Alam S. Sarker M. M. R. Afrin S. Richi F. T. Zhao C. Zhou J. R.. (2021). Traditional herbal medicines, bioactive metabolites, and plant products against COVID-19: update on clinical trials and mechanism of actions. Front. Pharmacol. 12:671498. doi: 10.3389/fphar.2021.671498, PMID: 34122096 PMC8194295

[ref5] Alassane-Kpembi I. Puel O. Pinton P. Cossalter A. M. Chou T. C. Oswald I. P. (2017). Co-exposure to low doses of the food contaminants deoxynivalenol and nivalenol has a synergistic inflammatory effect on intestinal explants. Arch. Toxicol. 91, 2677–2687. doi: 10.1007/s00204-016-1902-9, PMID: 27915442

[ref6] Bell M. A. Fischer R. A. (1994). Guide to plant and crop sampling: measurements and observations for agronomic and physiological research in small grain cereals. CIMMYT. Mexico.

[ref7] Berg S. Dennis P. G. Paungfoo-Lonhienne C. Anderson J. Robinson N. Brackin R.. (2020). Effects of commercial microbial biostimulants on soil and root microbial communities and sugarcane yield. Biol. Fertil. Soils 56, 565–580. doi: 10.1007/s00374-019-01412-4

[ref8] Bhattacharyya P. N. Jha D. K. (2012). Plant growth-promoting rhizobacteria (PGPR): emergence in agriculture. World J. Microbiol. Biotechnol. 28, 1327–1350. doi: 10.1007/s11274-011-0979-9 22805914

[ref9] Boulahouat S. Cherif-Silini H. Silini A. Bouket A. C. Luptakova L. Alenezi F. N.. (2023). Biocontrol efficiency of Rhizospheric *Bacillus* against the plant pathogen *fusarium oxysporum*: a promising approach for sustainable agriculture. Microbiol. Res. 14, 892–908. doi: 10.3390/microbiolres14030062

[ref10] Burlak O. P. De Vera J. P. Yatsenko V. Kozyrovska N. O. (2013). Putative mechanisms of bacterial effects on plant photosystem under stress. Вiopolym. Cell. 29, 3–10. doi: 10.7124/bc.000800

[ref11] Butterly C. R. Phillips L. A. Wiltshire J. L. Franks A. E. Armstrong R. D. Chen D.. (2016). Long-term effects of elevated CO_2_ on carbon and nitrogen functional capacity of microbial communities in three contrasting soils. Soil Biol. Biochem. 97, 157–167. doi: 10.1016/j.soilbio.2016.03.010

[ref12] Cai X. Zhao H. Liang C. Li M. Liu R. (2021). Effects and mechanisms of symbiotic microbial combination agents to control tomato fusarium crown and root rot disease. Front. Microbiol. 12:629793. doi: 10.3389/fmicb.2021.629793, PMID: 34220730 PMC8245789

[ref13] Chen J. Zhou L. Din I. U. Arafat Y. Li Q. Wang J.. (2021). Antagonistic activity of Trichoderma spp. against fusarium oxysporum in rhizosphere of Radix pseudostellariae triggers the expression of host defense genes and improves its growth under long-term monoculture system. Front. Microbiol. 12:579920. doi: 10.3389/fmicb.2021.579920, PMID: 33790872 PMC8005620

[ref14] Chou T. C. (2006). Theoretical basis, experimental design, and computerized simulation of synergism and antagonism in drug combination studies. Pharmacol. Rev. 58, 621–681. doi: 10.1124/pr.58.3.10, PMID: 16968952

[ref15] Deng Y. Han B. X. Hu D. J. Zhao J. Li S. P. (2018). Qualitation and quantification of water soluble non-starch polysaccharides from *Pseudostellaria heterophylla* in China using saccharide mapping and multiple chromatographic methods. Carbohydr. Polym. 199, 619–627. doi: 10.1016/j.carbpol.2018.06.063, PMID: 30143170

[ref16] Deng X. Zhang N. Li Y. Zhu C. Qu B. Liu H.. (2022). Bio-organic soil amendment promotes the suppression of *Ralstonia solanacearum* by inducing changes in the functionality and composition of rhizosphere bacterial communities. New Phytol. 235, 1558–1574. doi: 10.1111/nph.18221 35569105

[ref17] Ding J. Travers S. K. Eldridge D. J. (2022). Microbial communities are associated with indicators of soil surface condition across a continental gradient. Geoderma 405:115439. doi: 10.1016/j.geoderma.2021.115439

[ref18] Dukare A. Paul S. (2021). Biological control of *fusarium* wilt and growth promotion in pigeon pea (*Cajanus cajan*) by antagonistic rhizobacteria, displaying multiple modes of pathogen inhibition. Rhizosphere 17:100278. doi: 10.1016/j.rhisph.2020.100278

[ref19] Fernie A. R. Willmitzer L. (2001). Molecular and biochemical triggers of potato tuber development. Plant Physiol. 127, 1459–1465. doi: 10.1104/pp.010764, PMID: 11743089 PMC1540178

[ref20] Finkel O. M. Castrillo G. Paredes S. H. González I. S. Dangl J. L. (2017). Understanding and exploiting plant beneficial microbes. Curr. Opin. Plant Biol. 38, 155–163. doi: 10.1016/j.pbi.2017.04.018, PMID: 28622659 PMC5561662

[ref21] Finkel O. M. Salas-González I. Castrillo G. Conway J. M. Law T. F. Teixeira P. J. P. L.. (2020). A single bacterial genus maintains root growth in a complex microbiome. Nature 587, 103–108. doi: 10.1038/s41586-020-2778-7, PMID: 32999461 PMC10329457

[ref22] Foucquier J. Guedj M. (2015). Analysis of drug combinations: current methodological landscape. Pharmacol. Res. Perspect. 3:e00149. doi: 10.1002/prp2.149, PMID: 26171228 PMC4492765

[ref23] Gray E. J. Smith D. L. (2005). Intracellular and extracellular PGPR: commonalities and distinctions in the plant-bacterium signaling processes. Soil Biol. Biochem. 37, 395–412. doi: 10.1016/j.soilbio.2004.08.030

[ref24] Guo S. Jiao Z. Yan Z. Yan X. Deng X. Xiong W.. (2024). Predatory protists reduce bacteria wilt disease incidence in tomato plants. Nat. Commun. 15:829. doi: 10.1038/s41467-024-45150-0, PMID: 38280866 PMC10821857

[ref25] Guo S. Tao C. Jousset A. Xiong W. Wang Z. Shen Z.. (2022). Trophic interactions between predatory protists and pathogen-suppressive bacteria impact plant health. ISME J. 16, 1932–1943. doi: 10.1038/s41396-022-01244-5, PMID: 35461357 PMC9296445

[ref26] Hong Kong Chinese Materia Medica Standard (2020). Department of health: Hong Kong Special Administrative Region. The People’s Republic of China. Hong Kong SAR, China.

[ref27] Hu Z. Chen X. Yao J. Zhu C. Zhu J. Liu M. (2020). Plant-mediated effects of elevated CO_2_ and rice cultivars on soil carbon dynamics in a paddy soil. New Phytol. 225, 2368–2379. doi: 10.1111/nph.16298 31667850

[ref28] Hu J. Wei Z. Friman V. P. Gu S. H. Wang X. F. Eisenhauer N.. (2016). Probiotic diversity enhances rhizosphere microbiome function and plant disease suppression. MBio 7, e01790–e01716. doi: 10.1128/mbio.01790-16 27965449 PMC5156302

[ref29] Huo Y. Kang J. P. Park J. K. Li J. Chen L. Yang D. C. (2018). Rhodanobacter *ginsengiterrae* sp. nov., an antagonistic bacterium against root rot fungal pathogen *fusarium solani*, isolated from ginseng rhizospheric soil. Archi. Microbiol. 200, 1457–1463. doi: 10.1007/s00203-018-1560-9, PMID: 30116848

[ref30] Jia W. Zheng T. Zhao Y. Deng F. Yang Y. Liang C.. (2023). Nitrogen application influences the effect of bacteria on the belowground allocation of photosynthesized carbon under elevated CO_2_. Soil Biol. Biochem. 180:109021. doi: 10.1016/j.soilbio.2023.109021

[ref31] Jin J. Krohn C. Franks A. E. Wang X. Wood J. L. Petrovski S.. (2022). Elevated atmospheric CO_2_ alters the microbial community composition and metabolic potential to mineralize organic phosphorus in the rhizosphere of wheat. Microbiome 10:12. doi: 10.1186/s40168-021-01203-w, PMID: 35074003 PMC8785599

[ref32] Klapper M. Götze S. Barnett R. Willing K. Stallforth P. (2016). Bacterial alkaloids prevent amoebal predation. Angew. Chem. Int. Ed. 55, 8944–8947. doi: 10.1002/anie.201603312 27294402

[ref33] Kohler J. Caravaca F. del Mar Alguacil M. Roldán A. (2009). Elevated CO_2_ increases the effect of an arbuscular mycorrhizal fungus and a plant-growth-promoting rhizobacterium on structural stability of a semiarid agricultural soil under drought conditions. Soil Biol. Biochem. 41, 1710–1716. doi: 10.1016/j.soilbio.2009.05.014

[ref34] Kong H. G. Song G. C. Sim H. J. Ryu C. M. (2021). Achieving similar root microbiota composition in neighbouring plants through airborne signalling. ISME J. 15, 397–408. doi: 10.1038/s41396-020-00759-z, PMID: 32973341 PMC8027813

[ref35] Kramer-Walter K. R. Bellingham P. J. Millar T. R. Smissen R. D. Richardson S. J. Laughlin D. C. (2016). Root traits are multidimensional: specific root length is independent from root tissue density and the plant economic spectrum. J. Ecol. 104, 1299–1310. doi: 10.1111/1365-2745.12562

[ref36] Lasch A. Lichtenstein D. Marx-Stoelting P. Braeuning A. Alarcan J. (2020). Mixture effects of chemicals: the difficulty to choose appropriate mathematical models for appropriate conclusions. Environ. Pollut. 260:113953. doi: 10.1016/j.envpol.2020.113953, PMID: 31962267

[ref37] Lee S. H. Kang H. (2016). Elevated CO_2_ causes a change in microbial communities of rhizosphere and bulk soil of salt marsh system. Appl. Soil Ecol. 108, 307–314. doi: 10.1016/j.apsoil.2016.09.009

[ref38] Li Q. Li H. Yang Z. Cheng X. Zhao Y. Qin L.. (2022). Plant growth-promoting rhizobacterium *Pseudomonas* sp. CM11 specifically induces lateral roots. New Phytol. 235, 1575–1588. doi: 10.1111/nph.18199, PMID: 35510807 PMC9546010

[ref39] Li S. Xie S. Zhang S. Miao S. Tang S. Chen H.. (2022). Global patterns and controls of the soil microbial biomass response to elevated CO_2_. Geoderma 428:116153. doi: 10.1016/j.geoderma.2022.116153

[ref40] Liu Y. Chen L. Zhang N. Li Z. Zhang G. Xu Y.. (2016). Plant-microbe communication enhances auxin biosynthesis by a root-associated bacterium, *Bacillus amyloliquefaciens* SQR9. Mol. Plant-Microb. Interact. 29, 324–330. doi: 10.1094/MPMI-10-15-0239-R, PMID: 26808445

[ref41] Liu N. Shao C. Sun H. Liu Z. Guan Y. Wu L.. (2020). Arbuscular mycorrhizal fungi biofertilizer improves American ginseng (*Panax quinquefolius L*.) growth under the continuous cropping regime. Geoderma 363:114155. doi: 10.1016/j.geoderma.2019.114155

[ref42] Liu X. Wang H. Wu Y. Bi Q. Ding K. Lin X. (2022). Manure application effects on subsoils: abundant taxa initiate the diversity reduction of rare bacteria and community functional alterations. Soil Biol. Biochem. 174:108816. doi: 10.1016/j.soilbio.2022.108816

[ref43] Lozano G. L. Park H. B. Bravo J. I. Armstrong E. A. Denu J. M. Stabb E. V.. (2019). Bacterial analogs of plant tetrahydropyridine alkaloids mediate microbial interactions in a rhizosphere model system. Appl. Environ. Microbiol. 85, e03058–e03018. doi: 10.1128/AEM.03058-18, PMID: 30877115 PMC6498172

[ref44] Lugtenberg B. Kamilova F. (2009). Plant-growth-promoting rhizobacteria. Ann. Rev. Microbiol. 63, 541–556. doi: 10.1146/annurev.micro.62.081307.162918 19575558

[ref45] Ma Y. Shi L. Zhang Y. Gu H. Wang X. Dai L.. (2018). Effects of NAA on growth and active ingredients in root tuber of *Pseudostellaria heterophylla*. J. Nanjing For. Univ. 61:123. doi: 10.3969/j.issn.1000-2006.201701014

[ref46] Mamet S. D. Helgason B. L. Lamb E. G. McGillivray A. Stanley K. G. Robinson S. J.. (2022). Phenology-dependent root bacteria enhance yield of *Brassica napus*. Soil Biol. Biochem. 166:108468. doi: 10.1016/j.soilbio.2021.108468

[ref47] Mcgrath J. M. Lobell D. B. (2013). Reduction of transpiration and altered nutrient allocation contribute to nutrient decline of crops grown in elevated CO_2_ concentrations. Plant, Cell Environ. 36, 697–705. doi: 10.1111/pce.12007, PMID: 22943419

[ref48] Montealegre C. M. Van Kessel C. Russelle M. P. Sadowsky M. J. (2002). Changes in microbial activity and composition in a pasture ecosystem exposed to elevated atmospheric carbon dioxide. Plant Soil 243, 197–207. doi: 10.1023/A:1019901828483

[ref49] Nazari M. Smith D. L. (2020). A PGPR-produced bacteriocin for sustainable agriculture: a review of thuricin 17 characteristics and applications. Front. Plant Sci. 11:916. doi: 10.3389/fpls.2020.00916, PMID: 32733506 PMC7358586

[ref50] Ng C. W. W. Wang Y. C. Ni J. J. Tsim K. W. K. (2022a). Coupled effects of CO_2_ and biochar amendment on the yield and quality of *Pseudostellaria heterophylla*. Ind. Crop. Prod. 188:115599. doi: 10.1016/j.indcrop.2022.115599

[ref51] Ng C. W. W. Yan W. H. Tsim K. W. K. So P. S. Xia Y. T. To C. T. (2022b). Effects of *Bacillus subtilis* and *Pseudomonas fluorescens* as the soil amendment. Heliyon 8:e11674. doi: 10.1016/j.heliyon.2022.e11674, PMID: 36439778 PMC9691937

[ref52] Noctor G. Mhamdi A. (2017). Climate change, CO_2_, and defense: the metabolic, redox, and signaling perspectives. Trends Plant Sci. 22, 857–870. doi: 10.1016/j.tplants.2017.07.007, PMID: 28811163

[ref53] Oksanen J. Blanchet F. G. Friendly M. Kindt R. Legendre P. McGlinn D.. (2019). Package ‘vegan’. Community Ecology Package, version, 2(9).

[ref54] Ostonen I. Püttsepp Ü. Biel C. Alberton O. Bakker M. R. Lõhmus K.. (2007). Specific root length as an indicator of environmental change. Plant Biosyst. 141, 426–442. doi: 10.1080/11263500701626069

[ref55] Palaniyandi S. A. Yang S. H. Zhang L. Suh J. W. (2013). Effects of *Actinobacteria* on plant disease suppression and growth promotion. Appl. Microbiol. Biotechnol. 97, 9621–9636. doi: 10.1007/s00253-013-5206-1 24092003

[ref56] Paterson E. Hall J. M. Rattray E. A. S. Griffiths B. S. Ritz K. Killham K. (1997). Effect of elevated CO_2_ on rhizosphere carbon flow and soil microbial processes. Glob. Change Biol. 3, 363–377. doi: 10.1046/j.1365-2486.1997.t01-1-00088.x

[ref57] Pieterse C. M. J. Van Wees S. C. M. Ton J. Van Pelt J. A. Van Loon L. C. (2002). Signalling in rhizobacteria-induced systemic resistance in *Arabidopsis thaliana*. Plant Biol. 4, 535–544. doi: 10.1055/s-2002-35441

[ref58] Poorter H. Ryser P. (2015). The limits to leaf and root plasticity: what is so special about specific root length? New Phytol. 206, 1188–1190. https://www.jstor.org/stable/newphytologist.206.1188, doi: 10.1111/nph.13438 25952536

[ref59] Redondo-Gómez S. García-López J. V. Mesa-Marín J. Pajuelo E. Rodriguez-Llorente I. D. Mateos-Naranjo E. (2022). Synergistic effect of plant-growth-promoting rhizobacteria improves strawberry growth and flowering with soil salinization and increased atmospheric CO_2_ levels and temperature conditions. Agronomy 12:2082. doi: 10.3390/agronomy12092082

[ref60] Reich P. B. Hobbie S. E. Lee T. D. (2014). Plant growth enhancement by elevated CO_2_ eliminated by joint water and nitrogen limitation. Nat. Geosci. 7, 920–924. doi: 10.1038/ngeo2284

[ref61] Ren W. Liang P. Ma Y. Sun Q. Pu Q. Dong L.. (2021). Research progress of traditional Chinese medicine against COVID-19. Biomed. Pharmacother. 137:111310. doi: 10.1016/j.biopha.2021.111310, PMID: 33761591 PMC7857050

[ref62] Richardson A. E. Barea J. M. McNeill A. M. Prigent-Combaret C. (2009). Acquisition of phosphorus and nitrogen in the rhizosphere and plant growth promotion by microorganisms. Plant Soil 321, 305–339. doi: 10.1007/s11104-009-9895-2

[ref63] Roberts S. (2020). Quantifying genotypic and environmental factors affecting potato canopy growth. Doctoral dissertation, University of Cambridge

[ref64] Ruan Y. Kuzyakov Y. Liu X. Zhang X. Xu Q. Guo J.. (2023). Elevated temperature and CO_2_ strongly affect the growth strategies of soil bacteria. Nat. Commun. 14:391. doi: 10.1038/s41467-023-36086-y, PMID: 36693873 PMC9873651

[ref65] Sadowsky M. Schortemeyer M. (1997). Soil microbial responses to increased concentrations of atmospheric CO_2_. Glob. Change Biol. 3, 217–224. doi: 10.1046/j.1365-2486.1997.00078.x

[ref66] Šibanc N. Dumbrell A. J. Mandić-Mulec I. Maček I. (2014). Impacts of naturally elevated soil CO_2_ concentrations on communities of soil archaea and bacteria. Soil Biol. Biochem. 68, 348–356. doi: 10.1016/j.soilbio.2013.10.018

[ref67] Singh A. Siddappa S. Bhardwaj V. Singh B. Kumar D. Singh B. P. (2015). Expression profiling of potato cultivars with contrasting tuberization at elevated temperature using microarray analysis. Plant Physiol. Biochem. 97, 108–116. doi: 10.1016/j.plaphy.2015.09.014, PMID: 26447684

[ref68] Strange R. N. Scott P. R. (2005). Plant disease: a threat to global food security. Annu. Rev. Phytopathol. 43, 83–116. doi: 10.1146/annurev.phyto.43.113004.133839 16078878

[ref69] Sun X. Xu Z. Xie J. Hesselberg-Thomsen V. Tan T. Zheng D.. (2022). *Bacillus velezensis* stimulates resident rhizosphere *Pseudomonas stutzeri* for plant health through metabolic interactions. ISME J. 16, 774–787. doi: 10.1038/s41396-021-01125-3, PMID: 34593997 PMC8483172

[ref70] Tao C. Wang Z. Liu S. Lv N. Deng X. Xiong W.. (2023). Additive fungal interactions drive biocontrol of *fusarium* wilt disease. New Phytol. 238, 1198–1214. doi: 10.1111/nph.18793, PMID: 36740577

[ref71] van der Heul H. U. Bilyk B. L. McDowall K. J. Seipke R. F. van Wezel G. P. (2018). Regulation of antibiotic production in *Actinobacteria*: new perspectives from the post-genomic era. Nat. Prod. Rep. 35, 575–604. doi: 10.1039/C8NP00012C, PMID: 29721572

[ref72] Wei Z. Gu Y. Friman V. P. Kowalchuk G. A. Xu Y. Shen Q.. (2019). Initial soil microbiome composition and functioning predetermine future plant health. Sci. Adv. 5:eaaw0759. doi: 10.1126/sciadv.aaw0759, PMID: 31579818 PMC6760924

[ref73] Wei Z. Yang T. Friman V. P. Xu Y. Shen Q. Jousset A. (2015). Trophic network architecture of root-associated bacterial communities determines pathogen invasion and plant health. Nat. Commun. 6:8413. doi: 10.1038/ncomms9413, PMID: 26400552 PMC4598729

[ref74] Wei W. Ye C. Huang H. C. Yang M. Mei X. Y. Du F.. (2020). Appropriate nitrogen application enhances saponin synthesis and growth mediated by optimizing root nutrient uptake ability. J. Ginseng Res. 44, 627–636. doi: 10.1016/j.jgr.2019.04.003, PMID: 32617043 PMC7322810

[ref75] Williams A. Pétriacq P. Beerling D. J. Cotton T. A. Ton J. (2018). Impacts of atmospheric CO_2_ and soil nutritional value on plant responses to rhizosphere colonization by soil bacteria. Front. Plant Sci. 9:1493. doi: 10.3389/fpls.2018.01493, PMID: 30405655 PMC6204664

[ref76] Wu H. Qin X. Wang J. Wu L. Chen J. Fan J.. (2019). Rhizosphere responses to environmental conditions in *Radix pseudostellariae* under continuous monoculture regimes. Agri. Ecosyst. Environ. 270-271, 19–31. doi: 10.1016/j.agee.2018.10.014

[ref77] Wu D. X. Wang G. X. Bai Y. F. Liao J. X. (2004). Effects of elevated CO_2_ concentration on growth, water use, yield and grain quality of wheat under two soil water levels. Agri. Ecosyst. Environ. 104, 493–507. doi: 10.1016/j.agee.2004.01.018

[ref78] Wu H. Zhang Z. Wang J. Qin X. Chen J. Wu L.. (2021). Bio-fertilizer amendment alleviates the replanting disease under consecutive monoculture regimes by reshaping leaf and root microbiome. Microb. Ecol. 84, 452–464. doi: 10.1007/s00248-021-01861-1 34554283

[ref79] Xiao L. Liu G. B. Li P. Xue S. (2017). Effects of short-term elevated CO_2_ concentration and drought stress on the rhizosphere effects of soil carbon, nitrogen and microbes of *Bothriochloa ischaemum*. J. Appl. Ecol. 28, 3251–3259. doi: 10.13287/j.1001-9332.201710.009 29692143

[ref80] Xu F. Li C. Chen Y. Wu J. Bai H. Fan S.. (2024). Soil microbial community structure and soil fertility jointly regulate soil microbial residue carbon during the conversion from subtropical primary forest to plantations. Geoderma 441:116767. doi: 10.1016/j.geoderma.2023.116767

[ref81] Yadav J. Verma J. P. Jaiswal D. K. Kumar A. (2014). Evaluation of PGPR and different concentration of phosphorus level on plant growth, yield and nutrient content of rice (*Oryza sativa*). Ecol. Eng. 62, 123–128. doi: 10.1016/j.ecoleng.2013.10.013

[ref82] Yan W. H. Chen Y. L. Han L. F. Sun K. Song F. H. Yang Y.. (2022). Pyrogenic dissolved organic matter produced at higher temperature is more photoactive: insight into molecular changes and reactive oxygen species generation. J. Hazard. Mater. 425:127817. doi: 10.1016/j.jhazmat.2021.127817, PMID: 34883369

[ref83] Yang H. Fang C. Li Y. Wu Y. Fransson P. Rillig M. C.. (2022). Temporal complementarity between roots and mycorrhizal fungi drives wheat nitrogen use efficiency. New Phytol. 236, 1168–1181. doi: 10.1111/nph.18419, PMID: 35927946

[ref84] Yilmaz A. Karik Ü. (2022). AMF and PGPR enhance yield and secondary metabolite profile of basil (*Ocimum basilicum L*.). Ind. Crop. Prod. 176:114327. doi: 10.1016/j.indcrop.2021.114327

[ref85] Yu Z. Li Y. Wang G. Liu J. J. Liu J. D. Liu X. B.. (2016). Effectiveness of elevated CO_2_ mediating bacterial communities in the soybean rhizosphere depends on genotypes. Agric. Ecosyst. Environ. 231, 229–232. doi: 10.1016/j.agee.2016.06.043

[ref86] Yuan Q. S. Wang L. Wang H. Wang X. Jiang W. Ou X.. (2022). Pathogen-mediated assembly of plant-beneficial bacteria to alleviate *fusarium* wilt in *Pseudostellaria heterophylla*. Front. Microbiol. 13:842372. doi: 10.3389/fmicb.2022.842372, PMID: 35432244 PMC9005978

[ref87] Zak D. R. Pregitzer K. S. King J. S. Holmes W. E. (2000). Elevated atmospheric CO_2_, fine roots and the response of soil microorganisms: a review and hypothesis. New Phytol. 147, 201–222. doi: 10.1046/j.1469-8137.2000.00687.x

[ref88] Zhang F. Hou Y. Zed R. Mauchline T. H. Shen J. Zhang F.. (2023). Root exudation of organic acid anions and recruitment of beneficial actinobacteria facilitate phosphorus uptake by maize in compacted silt loam soil. Soil Biol. Biochem. 184:109074. doi: 10.1016/j.soilbio.2023.109074

[ref89] Zhang Y. Xiao J. Yang K. Wang Y. Tian Y. Liang Z. (2022). Transcriptomic and metabonomic insights into the biocontrol mechanism of *Trichoderma asperellum* M45a against watermelon *fusarium* wilt. PLoS One 17:e0272702. doi: 10.1371/journal.pone.0272702, PMID: 35947630 PMC9365129

[ref90] Zheng W. Zhou T. Li J. Jiang W. Zhang J. Xiao C.. (2019). The biosynthesis of Heterophyllin B in *Pseudostellaria heterophylla* from prePhHB-encoded precursor. Front. Plant Sci. 10:1259. doi: 10.3389/fpls.2019.01259, PMID: 31749814 PMC6842982

[ref91] Zytynska S. E. Eicher M. Rothballer M. Weisser W. W. (2020). Microbial-mediated plant growth promotion and pest suppression varies under climate change. Front. Plant Sci. 11:573578. doi: 10.3389/fpls.2020.573578, PMID: 33013998 PMC7511531

