# Supplementary material for: Plant growth-promoting rhizobacteria enhance active ingredient accumulation in medicinal plants at elevated CO2 and are associated with indigenous microbiome
Source: Front Microbiol. 2024 Aug 26;15:1426893. doi: 10.3389/fmicb.2024.1426893 (PMC11381388; doi:10.3389/fmicb.2024.1426893)
Supplement: Supplementary file 1 [file Data_Sheet_1.pdf]

Supplementary Material

1 Supplementary Figures and Tables

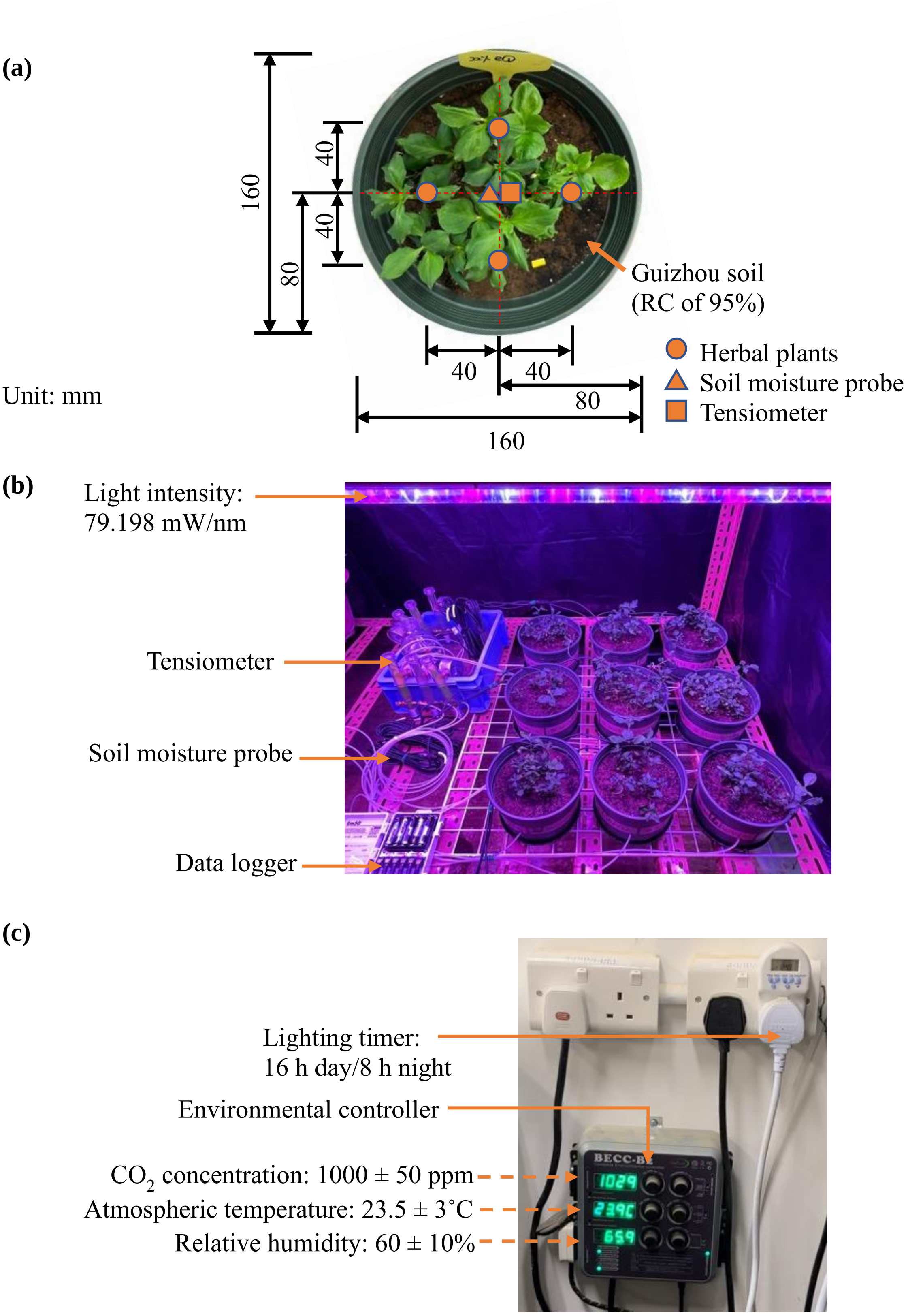

Fig. S1. An overview of the (a) pot set-up, (b) growth conditions, and (c) environmental controller.

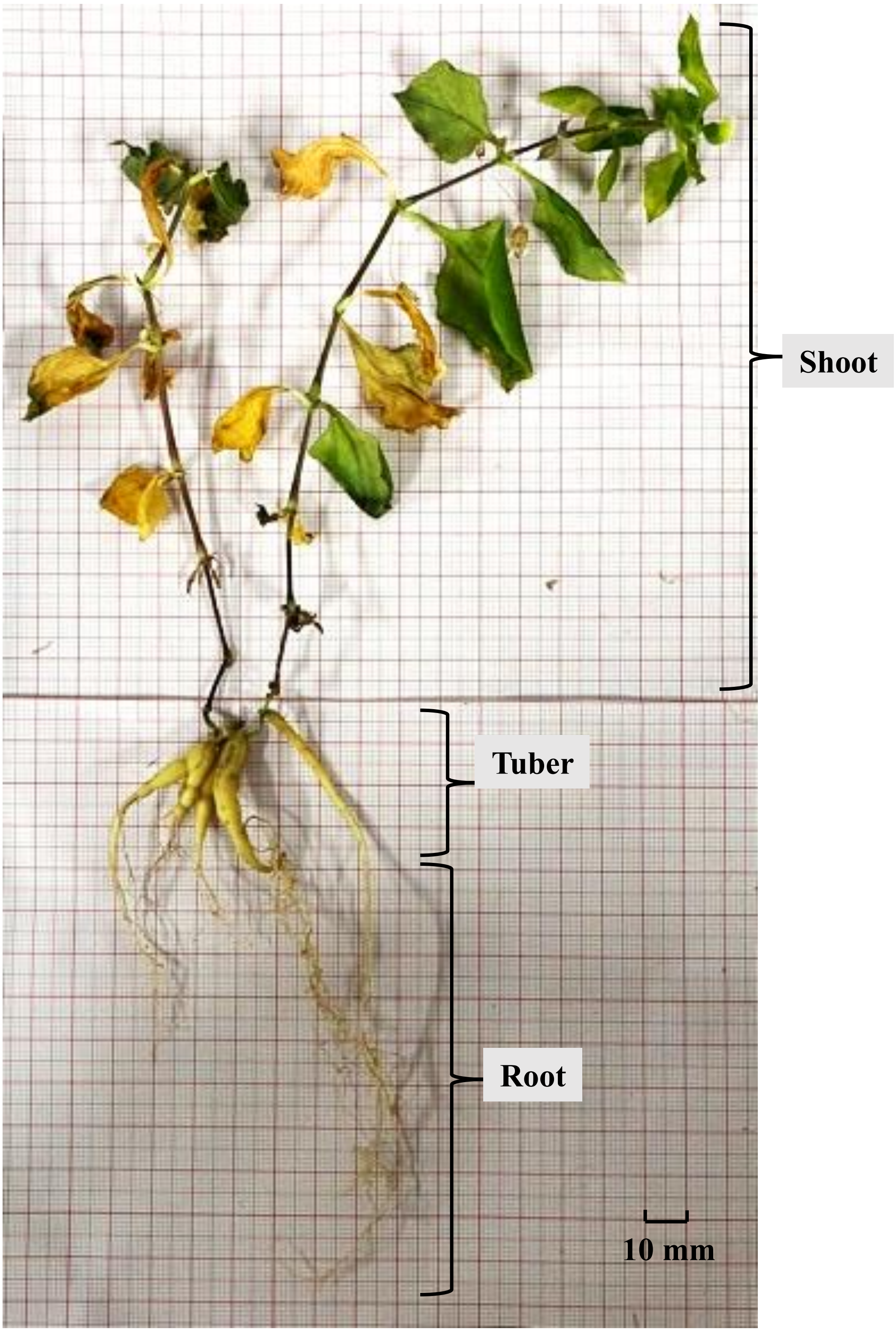

Fig. S2. An overview of a typical *Pseudostellaria heterophylla* after harvest.

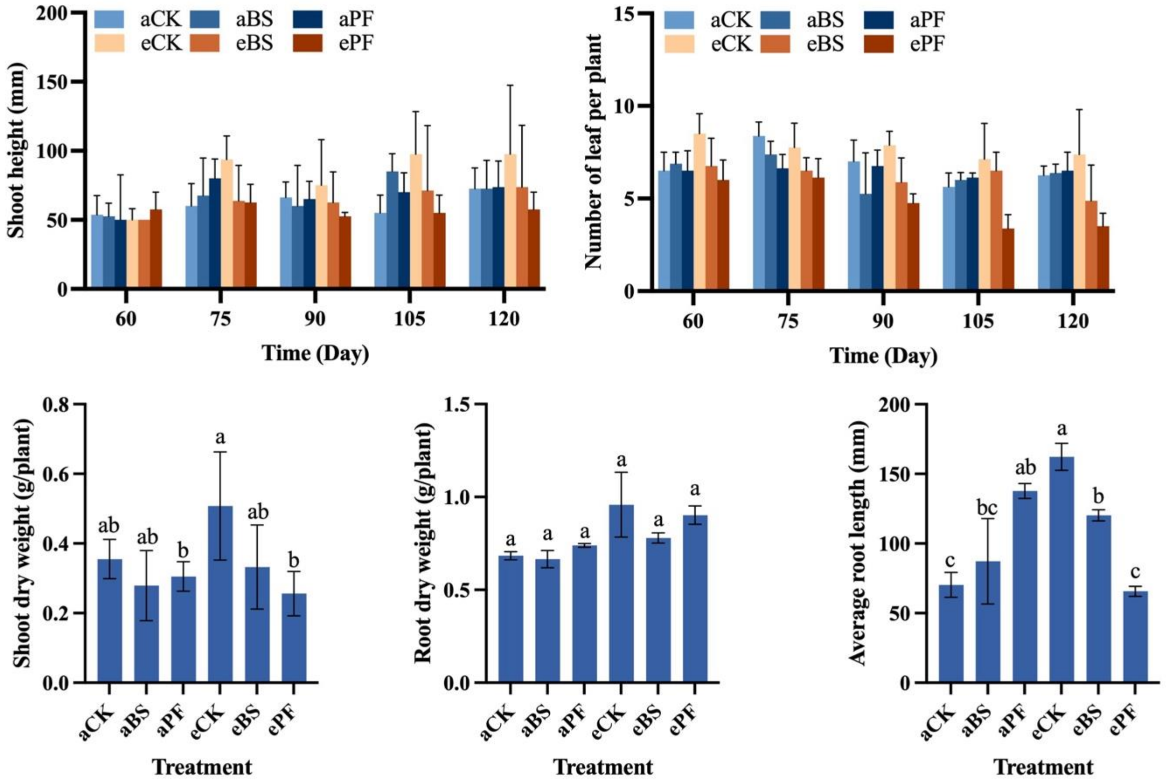

Fig. S3. Growth traits of *P. heterophylla* under different treatments. Abbreviations: a: ambient CO_2 condition (400 ppm). e: elevated CO_2 condition (1000 ppm). CK: control group without bacterial inoculation. BS: *Bacillus subtilis*-inoculated group. PF: *Pseudomonas fluorescence*-inoculated group. Bars represent the mean with standard deviation (n = 12-19). Different letters on the top of each bar indicate the significance of differences among treatments ($p < 0.05$).

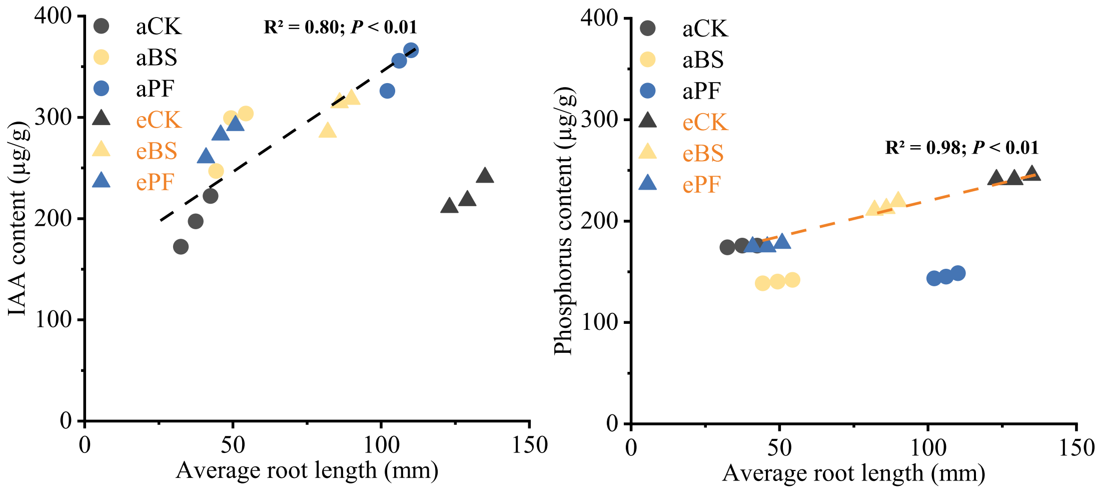

Fig. S4. Relationship between average root length and the content of indole-acetic acid (IAA) or phosphorus in rhizosphere soil. Abbreviations: a: ambient CO_2 condition (400 ppm). e: elevated CO_2 condition (1000 ppm). CK: control group without bacterial inoculation. BS: *Bacillus subtilis*-inoculated group. PF: *Pseudomonas fluorescence*-inoculated group. Bars represent the mean with standard deviation (n = 12-19). Different letters on the top of each bar indicate the significance of differences among treatments ($p < 0.05$).

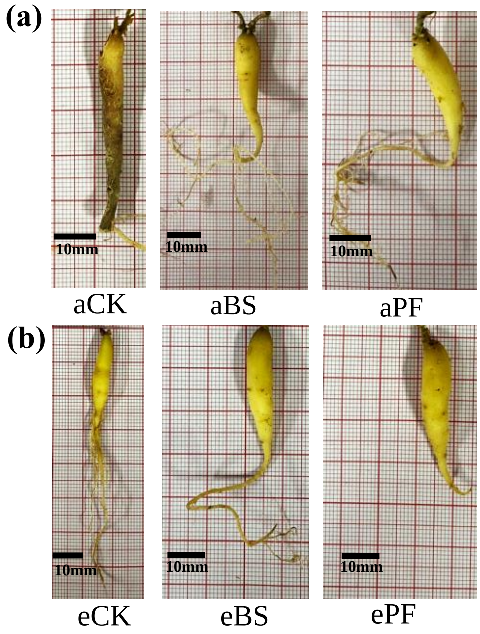

Fig. S5. Tubers of *P. heterophylla* subjected to different treatments after harvest. (a) Ambient CO_2 condition. (b) Elevated CO_2 condition. Abbreviations: a: ambient CO_2 condition (400 ppm). e: elevated CO_2 condition (1000 ppm). CK: control group without bacterial inoculation. BS: *Bacillus subtilis*-inoculated group. PF: *Pseudomonas fluorescence*-inoculated group.

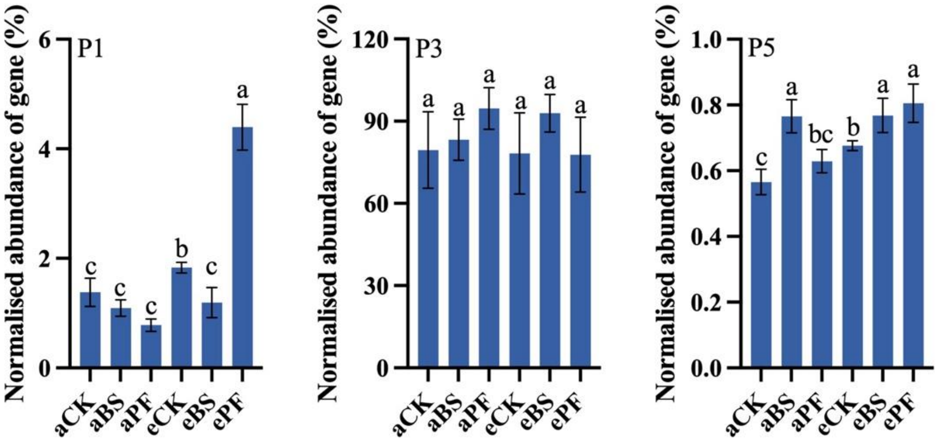

Fig. S6. KEGG analysis of the normalised abundance of genes in response to PGPR and eCO_2. P1: Biosynthesis of macrolides. P3: Biosynthesis of various other antibiotics. P5: Clavulanic acid biosynthesis. Bars in (B) represent the mean with standard deviation (n = 3). Different letters on the top of each bar indicate the significance of differences among treatments ($p < 0.05$).

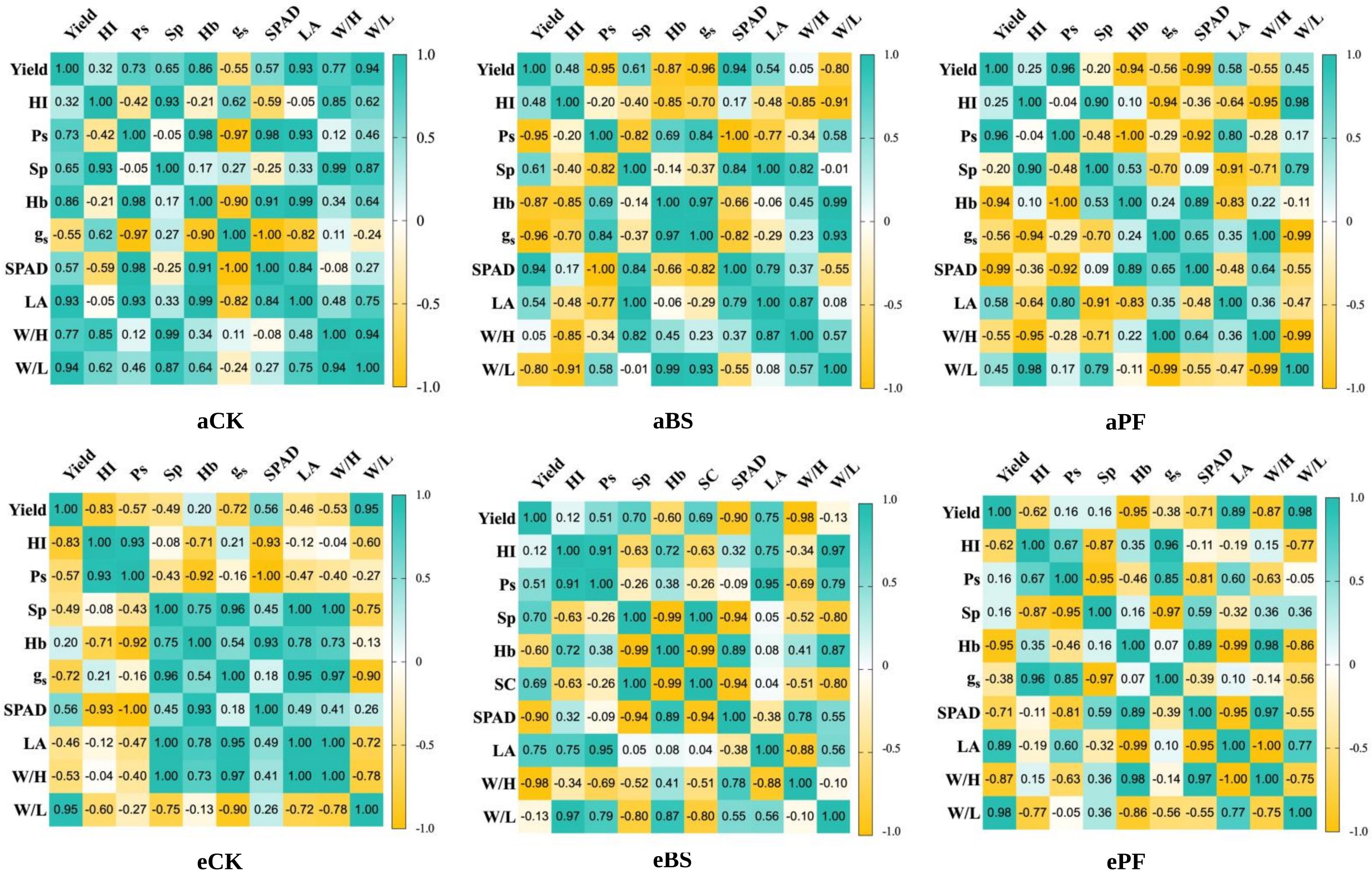

Fig. S7. Pearson correlation analysis among different traits of *P. heterophylla* individuals cultivated with inoculation of PGPR (*B. subtilis* and *P. fluorescens*) under different levels of atmospheric CO_2 (ambient: 400 ppm, elevated: 1000 ppm). Harvest index (HI); Polysaccharides (Ps); Saponins (Sp); Heterophyllin B (Hb); Stomatal conductance (g_s); Soil plant analysis development (SPAD); Leaf area (LA); Ratio of shoot dry weight to height (W/H); Ratio of root dry weight to length (W/L), $p<0.05$.

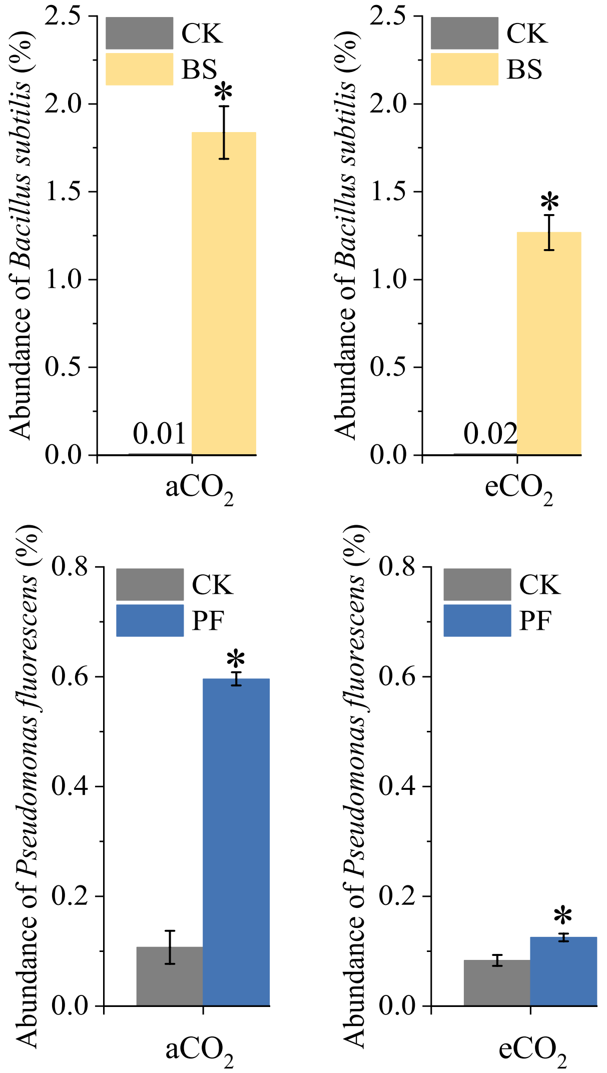

Fig. S8. Effects of atmospheric CO_2 on the colonisation of *Bacillus subtilis* and *Pseudomonas fluorescens.* CK: control group without bacterial inoculation. BS: *Bacillus subtilis*-inoculated group. PF: *Pseudomonas fluorescens*-inoculated group. aCO_2: ambient CO_2 concentration (400 ppm). eCO_2: elevated CO_2 concentration (1000 ppm). Bars represent the mean with standard deviation (n = 3). * represents a significant difference between treatments ($p < 0.05$).

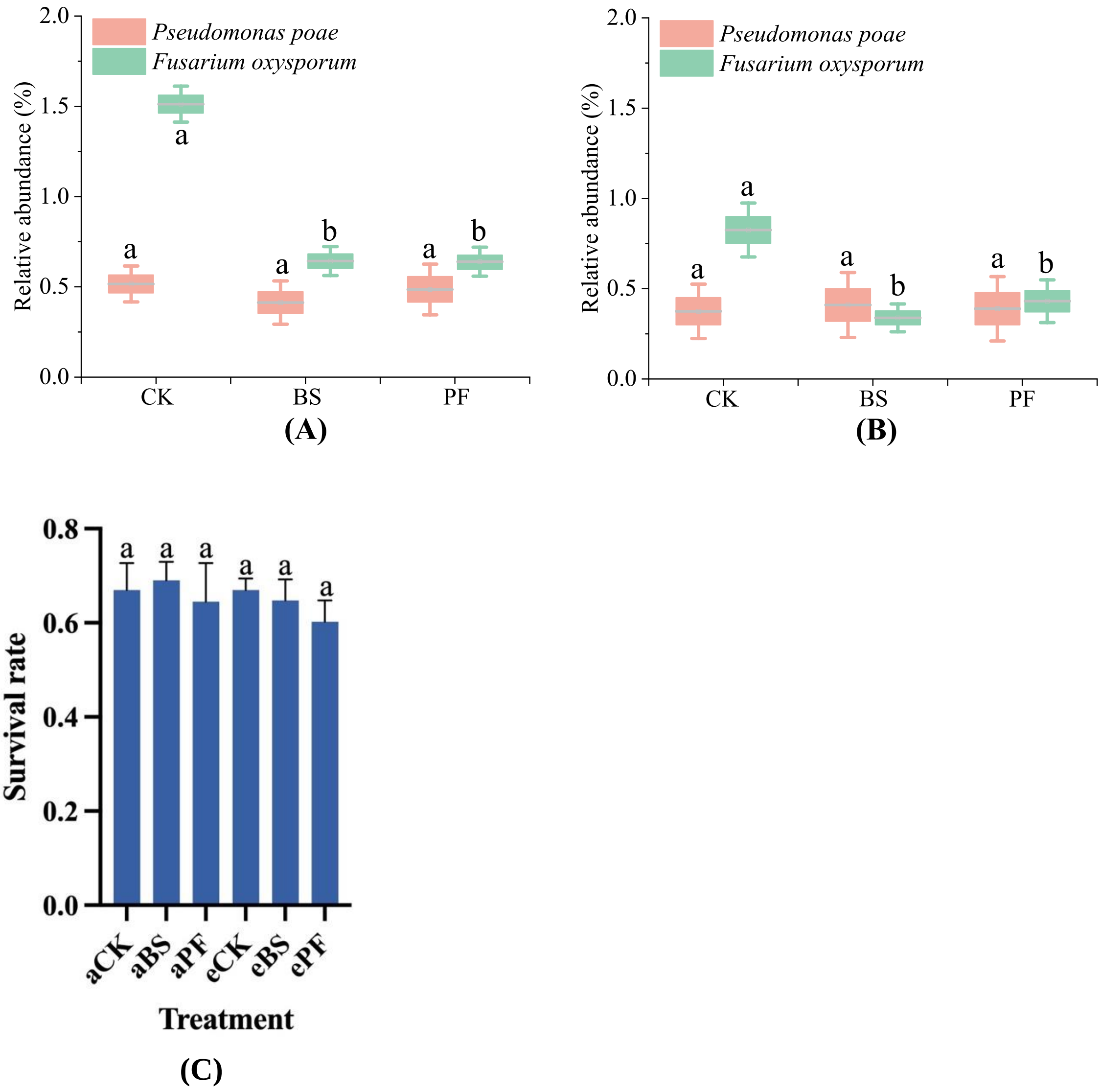

Fig. S9. Relative abundance of disease-associated microbes at aCO_2 (A) and eCO_2 (B) as well as plant survival rate (C). aCO_2 condition (400 ppm). eCO_2 condition (1000 ppm). CK: control group without bacterial inoculation. BS: *Bacillus subtilis*-inoculated group. PF: *Pseudomonas fluorescens*-inoculated group. Boxes represent the mean with standard deviation (n = 3). Different letters on the top of the boxes indicate the significance of differences among treatments ($p < 0.05$), n=3.

Table S1. Tuber morphology of *P. heterophylla*

Treatment	Length (mm)	Maximum width (mm)
aCK	34.19 ± 2.88a	6.12 ± 0.58b
aBS	42.11 ± 12.10a	7.53 ± 0.21a
aPF	39.44 ± 7.88a	7.67 ± 0.52a
eCK	31.42 ± 2.71a	7.35 ± 0.91a
eBS	29.44 ± 2.51a	6.17 ± 1.66ab
ePF	25.00 ± 0.25b	7.33 ± 0.46a

Note:
Abbreviations: a: ambient CO_2 condition (400 ppm). e: elevated CO_2 condition (1000 ppm). CK: control group without bacterial inoculation. BS: *Bacillus subtilis*-inoculated group. PF: *Pseudomonas fluorescence*-inoculated group. Data presented are the mean ± standard deviation (n = 15-20). Different letters beside data indicate the significance of differences among treatments ($p < 0.05$).

Table S2. Species alpha diversity analysis of rhizosphere soil

Treatment	Chao 1 index	Shannon index	Simpson index
aCK	5057 ± 71b	6.88 ± 0.47a	0.99 ± 0.02a
aBS	5098 ± 54b	6.80 ± 0.39a	0.99 ± 0.01a
aPF	5008 ± 50b	6.85 ± 0.37a	0.99 ± 0.01a
eCK	5243 ± 85a	6.80 ± 0.10a	0.99 ± 0.01a
eBS	5061 ± 92b	6.72 ± 0.35a	0.99 ± 0.01a
ePF	5005 ± 73b	6.70 ± 0.44a	0.99 ± 0.01a

Note:
Abbreviations: a: ambient CO_2 condition (400 ppm). e: elevated CO_2 condition (1000 ppm). CK: control group without bacterial inoculation. BS: *Bacillus subtilis*-inoculated group. PF: *Pseudomonas fluorescence*-inoculated group. Data presented are the mean ± standard deviation (n = 3). Different letters beside data indicate the significance of differences among treatments ($p < 0.05$).
